# Tension and Robustness in Multitasking Cellular Networks

**DOI:** 10.1371/journal.pcbi.1002491

**Published:** 2012-04-26

**Authors:** Jeffrey V. Wong, Bochong Li, Lingchong You

**Affiliations:** 1 Department of Biomedical Engineering, Duke University, Durham, North Carolina, United States of America; 2 Institute for Genome Sciences and Policy, Duke University, Durham, North Carolina, United States of America; 3 Center for Systems Biology, Duke University, Durham, North Carolina, United States of America; North Carolina State University, United States of America

## Abstract

Cellular networks multitask by exhibiting distinct, context-dependent dynamics. However, network states (parameters) that generate a particular dynamic are often sub-optimal for others, defining a source of “tension” between them. Though multitasking is pervasive, it is not clear where tension arises, what consequences it has, and how it is resolved. We developed a generic computational framework to examine the source and consequences of tension between pairs of dynamics exhibited by the well-studied RB-E2F switch regulating cell cycle entry. We found that tension arose from task-dependent shifts in parameters associated with network modules. Although parameter sets common to distinct dynamics did exist, tension reduced both their accessibility and resilience to perturbation, indicating a trade-off between “one-size-fits-all” solutions and robustness. With high tension, robustness can be preserved by dynamic shifting of modules, enabling the network to toggle between tasks, and by increasing network complexity, in this case by gene duplication. We propose that tension is a general constraint on the architecture and operation of multitasking biological networks. To this end, our work provides a framework to quantify the extent of tension between any network dynamics and how it affects network robustness. Such analysis would suggest new ways to interfere with network elements to elucidate the design principles of cellular networks.

## Author Summary

Multitasking pervades our daily lives. For example, the technological devices that we increasingly rely upon are now engineered with such multifunctionality or “integration” in mind. Similarly, cellular networks also multitask in that they generate multiple, distinct dynamics according to their operating context. Here we show that differences in parameter spaces that underlie different dynamics thus cause a “tension”, which ultimately constrains network operation. In particular, our analysis reveals that tension negatively impacts robustness by reducing accessibility of parameters able to accomplish two tasks and reduces their ability to withstand perturbations. The presence of tension and its negative impact on network robustness represents a fundamental, generic constraint on the operation of different multitasking networks.

## Introduction

**Figure 1:**
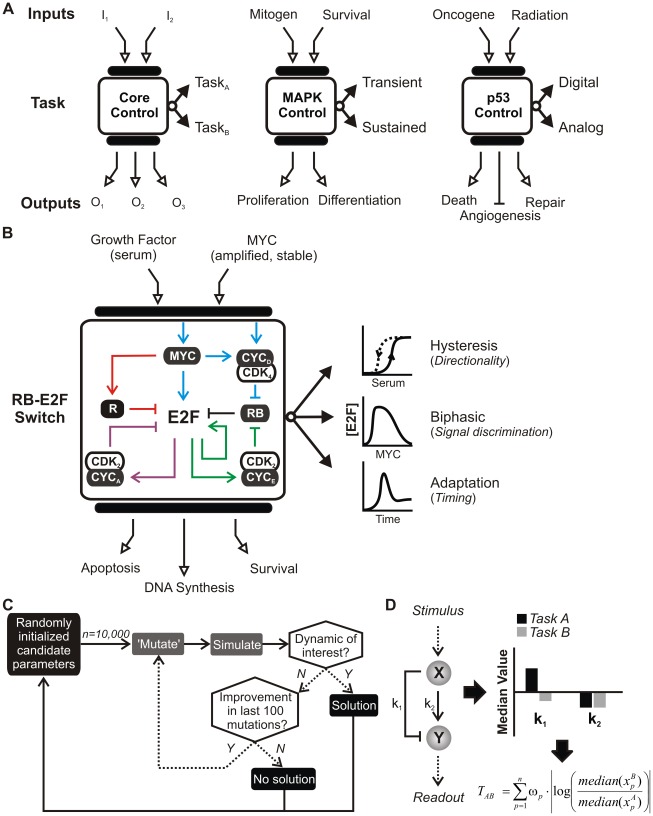
Multitasking networks. *(A)* (*Left*) A generic pathway consisting of system inputs (I); upstream and downstream mediators (black bars); a core network; and outputs (O). (*Middle*) The mitogen-activated protein kinase (MAPK) cascade mediates a myriad of growth factor signals and elicits, sometimes opposing responses in a cell-type specific manner. (*Right*) The p53 network responds to a variety of cell stresses and elicits an appropriate course of gene expression that mitigates uncontrolled proliferation. *(B)* The RB-E2F switch, partitioned into four modules: Sensor (cyan edges); repression (R, red); negative feedback (NFB, purple); and positive feedback (PFB, green). This pathway performs at least three dynamic tasks in response to growth signals. *(C)* Algorithmic approach to search parameter space. *(D)* Calculating tension. Given a network, solution sets of parameters able to generate each dynamic (A and B) are identified. Tension between tasks (T_AB_) is defined as the weighted sum of the log-ratio of median parameter values.

Decades of experimental studies have established detailed “wiring diagrams” of diverse cellular networks. A striking property of many networks is multitasking – the ability to generate different dynamics according to their operating context (Figure 1*A*). For example, the mitogen-activated protein kinase (MAPK) pathway involving RAF-MEK-ERK responds to epidermal growth factor (EGF) by triggering transient ERK activation in a graded fashion whereas nerve growth factor (NGF) induced sustained ERK in bistable manner [1]. These tasks directly underlie contrasting biological outcomes: EGF induces proliferation whereas NFG induces differentiation into neurons. Another example concerns the p53 stress response network that mediates arrest, death, and DNA repair functions [2]. In response to ionizing radiation, the network generates multiple pulses of p53 with constant amplitude (i.e. digital) [3] whereas UV-radiation generates a single, broad pulse whose amplitude follows a graded dose-response (i.e. analog) [4]. Insight into how distinct p53 tasks translate into biological outcomes is just beginning to emerge [5].

Multitasking networks are speculated to have arisen through successive elaboration on pre-existing “core” processes, representing an evolutionarily feasible route to generate novel biological attributes [6]. Intuitively, reusing a common set of components to multitask can be an economical way to accomplish multiple biological goals. Yet, such a strategy can pose an operational challenge: A dynamic may require network states (each being defined by a set of parameter values) that are ill suited for other dynamics. This concept is related to applications of multi-objective optimization (MOO) algorithms in engineering [7], where two or more, possibly conflicting design aspects are considered. Recently, these approaches have been adopted for biology in problems involving classification, system optimization, and gene regulatory network inference [8]. Here, we use “tension” to describe the difference in parameter spaces for distinct dynamics. Intuitively, tension increases with the number of tasks that a network is charged with as each task invariably requires a different subset of parameter values. In the extreme, tension can constrain a network to the point that few additional changes to the network can be tolerated.

A full understanding of network design principles requires an appreciation of where such tensions can arise within networks, their consequences on the robustness of each dynamic, and the strategies used to overcome them. Thus far, however, such concepts have been neglected in quantitative analysis of natural and synthetic pathways. To this end, we have developed a generic computational framework to allow streamlined examination of these questions. We illustrate the use of this framework by analyzing a well-studied RB-E2F network, which plays a pivotal role in regulating cell cycle entry.

## Results

### The multitasking RB-E2F network

The RB-E2F network has been examined in detail under both normal [9] and pathological [10] circumstances (Figure 1*B* and supporting text (Text S1)). In quiescent cells, E2F is silenced by RB [9] whereas E2F expression and activity is modulated by growth stimulation through four “modules” – interconnected subsets of the network with a distinct regulatory effect. A *sensor module* links extracellular growth stimulation and E2F activity: Upon growth stimulation, the MYC level increases and facilitates E2F expression [11] directly and via D-type Cyclins (CYC_D_), which potentiate kinases (CDK_4/6_) to inactivate RB. A *positive feedback module* (**PFB**) reinforces E2F expression by two routes: E2F can bind to its own promoter and maintain an activated state [12] and E2F increases expression of Cyclin E (CYC_E_) [13], which activates another RB-kinase (CDK_2_). A *negative feedback module* (**NFB**) consists of E2F-regulated genes that include Cyclin A [14] and SKP2 [15] that inactivate E2F binding and induce proteolysis, respectively. Finally, a *repression module* (**R**) consists of MYC-regulated genes that down-regulate E2F expression, which may include microRNAs within the *miR-17-92* cluster [16] and the ARF tumor suppressor [17].

Three distinct E2F dynamics underlie the response to growth stimuli, depending on the operating context of the network. First, E2F is *bistable* with respect to serum. Once activated, E2F remains ON even if serum is reduced below the threshold required to activate E2F [18]. In particular, the serum response of E2F exhibits hysteresis, whereby activation of E2F from the OFF state (by increasing serum) and shutting-OFF from the ON state (by decreasing serum) follow different trajectories (Figure 1*B*). This property provides a mechanism for cells to enforce two distinct states, quiescence and proliferation [19]: Cells will commit to the cell cycle when a growth stimulus exceeds an activation threshold and to quiescence when signals drop below a maintenance threshold.

Second, E2F exhibits *biphasic response* to direct MYC stimulation: E2F expression increases with the MYC level when the latter is low, but is repressed when the MYC level is too high [20]. This response restricts the range of MYC levels that can activate E2F. It may represent a safeguard mechanism that allows cells to distinguish physiological levels of MYC induced by serum from transient, potentially oncogenic levels resulting from gene mutation or stochastic gene expression.

Third, in normal cells strongly stimulated by serum, E2F expression exhibits *temporal adaptation*: It increases to a high level leading up to the end of G_1_ before being down-regulated as cells enter the S-phase [21]. As E2F controls expression of many genes involved in DNA synthesis [22], adaptive E2F can both promote coherent induction of DNA replication activities and restrict them to a brief period in S-phase. Indeed, precocious or prolonged E2F activity has been shown to cause replicative stress resulting from deregulated DNA synthesis followed by a DNA damage checkpoint [23], [24].

### Modeling framework

The starkly different dynamics generated by the same network led us to hypothesize the existence of conflicts that constrain its operation. To examine this issue, we probed several questions by modeling: How (dis)similar are the solution set of parameters that underlie different dynamics? What is the relative difficulty in identifying such parameter sets and what properties do they demonstrate in terms of network performance? In short, for a specific set of dynamics, what is the relationship between tension and robustness?

Here, we have developed a generic computational approach to examine these questions (Figure 1*C*). Candidate parameter sets were used to simulate from the model and assigned a score based upon an objective function (Figure S1 *A*). In a single iteration of the algorithm, randomly initialized parameter sets were subjected to successive rounds of ‘mutation’ followed by scoring. If a solution was identified, the iteration was terminated or it was terminated without a solution after a defined number of consecutive mutations (in this case 100) without an improvement in the objective score.

This analysis allowed us to enumerate parameter sets that satisfy each particular task (i.e. single) or biologically relevant pairings (i.e. dual). For two tasks (e.g., A and B), tension is calculated as the weighted sum of the log-ratio of median parameter values (Figure 1*D*). In the case that each parameter receives equal weighting (i.e. 1/n, where n is the number of free parameters), tension is the average extent each parameter shifts between single tasks. We evaluate robustness according to the “accessibility” of dual solutions and “resilience” of single-task or dual solutions to parameter perturbation. Accessibility is defined as the fraction of single-task solutions identified as dual. A decrease in accessibility indicates increasing difficulty in locating dual solutions. Resilience is defined as the ability of a solution to maintain some minimal performance after a perturbation (in this case at least 10% of the objective score). This framework can be applied to any kinetic model of cellular networks where objective functions can be quantified.

### Tension and coordination between bistable and biphasic responses

**Figure 2:**
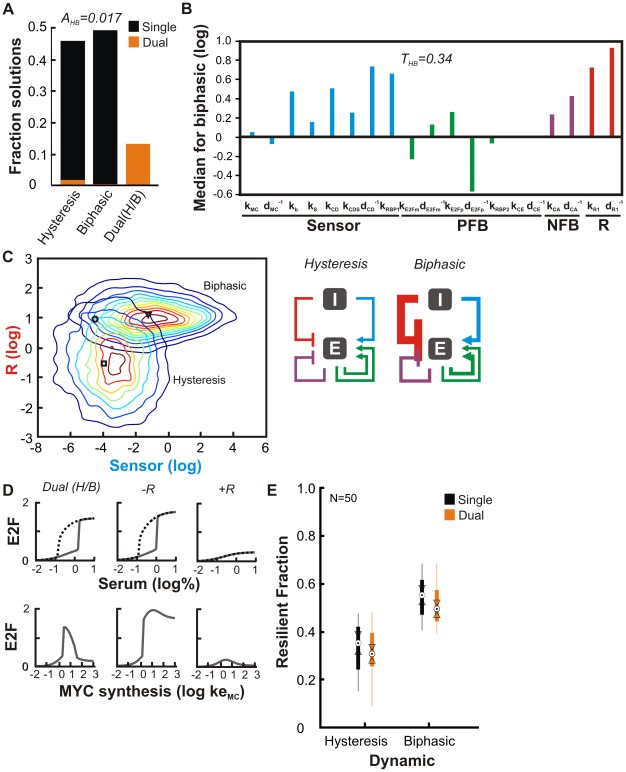
Tension between hysteresis and biphasic dose-response. *(A)* Fraction of parameter sets resulting from 10,000 algorithm iterations that generated hysteretic (n = 4,541), biphasic (n = 4,878) or dual dynamics (Dual(H/B); n = 1,290). Orange region within results for hysteresis and biphasic are the subset that support dual behavior. Hysteresis is measured by calculating the path difference 24 hours after an increase in serum from 0.01% or after a decrease from 10%. Biphasic response is measured 36 hours after an increase in MYC synthesis (parameter ke_MC_; Text S1). *(B)* Network state for biphasic behavior. Median value of parameters that support biphasic dose-response using hysteresis as the baseline (i.e. zero value). Related parameters were grouped into individual modules. *(C)* (*Left*) Distribution of solution module strengths (product over all parameters) for solutions to hysteresis and biphasic behavior. Also indicated are median values for hysteresis (square), biphasic response (triangle), and Dual(H/B) (circle) dynamics. (*Right*) Coordination of hysteresis and biphasic behavior. Relative module strengths are indicated by line thickness. I – growth inputs from serum and MYC; E – E2F. *(D)* Simulations of a representative Dual(H/B) solution in the vicinity of the median. R module strength was modulated by replacing it with median value from solutions for hysteresis (−R) or biphasic response (+R). E2F is expressed in µM. *(E)* Resilience of individual solutions. Boxplots summarize the resilience of 50 solutions for hysteresis, biphasic, and Dual (H/B). Solutions were selected by identifying the smallest box (values of sensor and R) centered on the median containing 50 solutions. Resiliency is defined as the ability to maintain at least 10% of their objective score following parameter perturbation. Y-axis shows the fraction of 10,000 repeated perturbations to a particular solution that are resilient. Circle indicates median; Medians are significantly different at the 5% significance level if there is no overlap between intervals defined by their triangular notches.

We first compared the bistable response to serum and the biphasic response to MYC. From 10,000 iterations we identified a large fraction of solutions for each single task (Figure 2*A*). However, only 146 dual solutions were present amongst 4,541 for hysteresis and 14 dual solutions were present in the 4,878 for biphasic. This result corresponds to a dual-solution accessibility of A_HB_ = 0.017, that is, dual solutions represent 1.7% of the total. The rate of solutions identified per iteration and dual accessibility was similar even when only 500 iterations were performed (Figure S1 *B*), indicating that the result from 10,000 iterations is representative.

Reduced accessibility may reflect tension in the network that arises because single dynamics may adopt disparate states. To examine the correlation between shifts in dynamics and corresponding changes in parameters, we determined the median value of each parameter from all the solutions. By using the values for hysteresis as a reference, we isolated changes specifically associated with biphasic response. According to their influence on each module (i.e., synthesis rates are proportional whereas degradation constants are inversely proportional to module strength), parameters were grouped into four modules (sensor, NFB, PFB, and R). This analysis identified biases in the solution parameters associated with sensor, NFB, and R modules (Figure 2*B*), whereas changes to the PFB parameters were divergent (see supporting Text S1, Discussion). Note that the overall distribution of NFB values were quite similar between different dynamics despite a change in median (Figure S2 *A*). The changes across all median parameter values resulted in a tension of 0.34 (i.e. average shift in parameter value) between hysteretic and biphasic tasks.

Parameter distributions may be highly irregular, raising the issue of how the median may perform as a summary of each solution set. An alternative approach to compare distributions is to calculate the Kullback–Leibler (KL) divergence (Text S1). Consistent with the results obtained using median values, the largest KL divergence involved parameters of R (Figure S2 *B*). More subtle distances in NFB, Sensor, and NFB were also present. In this case, the tension value (0.08) was calculated as the average KL divergence. All subsequent analyses were done by using the median values.

The difference between the two dynamics can be largely accounted for by the strength of sensor and R modules - the product of free parameters constituting each module (Figure 2*C*). Given this observation, an effective strategy to reconcile the tension is to dynamically configure these modules: Increasing their strengths would favor biphasic response, while decreasing them would favor hysteresis. In contrast, changes in other modules would be less critical. We term this dynamical ‘network reconfiguration’.

The overlap between R and sensor (Figure 2*C*), however, also suggests the possibility to accommodate the two dynamics by using common parameter sets, which, by definition, represent dual solutions. We performed 10,000 search iterations using a composite objective function that represents the product of hysteretic and biphasic objectives (see Text S1, Materials and Methods) which allowed us to identify an additional 1,290 dual solutions (Figure 2*A*). Most dual solutions were concentrated in the overlap between single-solution sets, consistent with the notion that they represent a hybrid of parameters from single dynamics (Figure S2 *C*). To validate the distribution of these dual solutions, we also attempted a search using a dual objective function composed of the sum of individual objectives. In addition, we performed a search with single hysteretic and biphasic solutions as a starting point, mimicking the successive elaboration of network tasks. In each case, the distribution of solutions parameters was virtually indistinguishable (Figure S2 *A* and *C*). This supports the notion that the distribution of dual solutions is representative.

Simulations show that a typical dual solution could indeed generate both dynamics (Figure 2*D*). Consistent with Figure 2*C*, weakening the R module (by substituting it with the median value from hysteretic solutions) diminished the repression of E2F at high MYC, thus diminishing the biphasic response. In contrast, strengthening the R module (by substituting it with the median value from biphasic solutions) maintained the biphasic response to MYC but eliminated hysteresis by weakening overall E2F response. Weakening the sensor shifted the hysteretic response to higher serum inputs but diminished the E2F levels achieved in response to MYC (Figure S2 *D*); strengthening the sensor eliminated hysteresis and broadened the biphasic response by stimulating an increase in E2F at relatively low doses of input.

A caveat of such dual solutions is their reduced accessibility (Figure 2*A*). In addition, it is interesting to examine if tension could also impact their resiliency to perturbation. To examine this, we selected fifty representative solutions from each category in the vicinity of their respective medians, subjected each one to 10,000 parameter perturbations, and determined the fraction of perturbations that retained at least 10% of the initial objective score. This analysis revealed that biphasic response was a more resilient property than hysteresis overall (Figure 2*E* and Figure S2 *E*).

Although the median resiliency of dual solutions was slightly lower than single solutions, this change was not significant, suggesting this tension had a minimal impact on the performance of dual solutions. As such, properly configured sensor and R modules can accommodate both dynamics. This could be achieved by engaging the R module only when MYC is sufficiently high, yet simultaneously enhancing the sensitivity of E2F to MYC stimulation. This notion is consistent with the distinct modes of MYC regulation in physiological and pathological contexts. Physiological stimulation, e.g., by serum, of arrested cells leads to a pulse of MYC that drops to a low level throughout the cell cycle [11], which is unable to trigger the R module. Still, a strong sensor module would enable robust generation of E2F switching behavior despite relatively low MYC levels (second column of Figure 2*D*). In contrast, more elevated and persistent levels of MYC, due to overexpression or stochastic gene expression, would trigger the R module and result in biphasic response.

### Tension and coordination between hysteretic and adaptive responses

**Figure 3:**
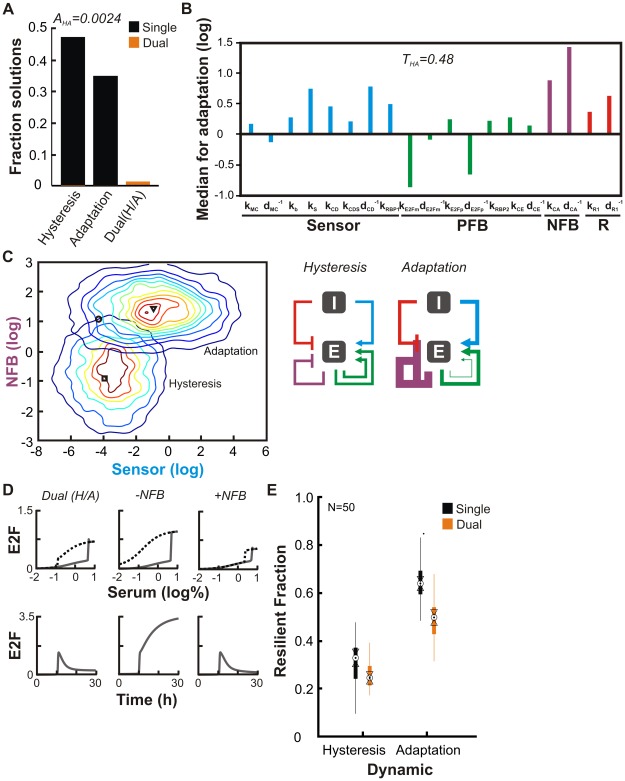
Tension between hysteresis and adaptive response. *(A)* Fraction of parameter sets resulting from 10,000 algorithm iterations that generated hysteresis (n = 4,541 solutions), adaptation (n = 3,353) or dual dynamics (n = 105). Temporal adaptation occurs in response to a shift in serum from 0.01% to 10%. *(B)* Network state for adaptation. *(C)* (*Left*) Distribution of module strengths for solutions to hysteresis and adaptation. Also indicated are median values for solutions to hysteresis (square), adaptation (triangle), and dual (circle) dynamics. (*Right*) Coordination of hysteresis and adaptation. (*D*) Simulations of a representative Dual(H/A) solution in the vicinity of the median (circle in Figure 3C). The NFB strength was modulated by replacing it with median values from hysteresis (−NFB) or adaptation (+NFB). For serum response, solid lines represent levels 24 hours after an increase from 0.01% serum; dotted lines are levels when initial conditions were 10% serum. E2F is expressed in µM. *(E)* Resiliency of solutions in the vicinity of the median for hysteresis, adaptation, and Dual (H/A) data. Each boxplot summarize the results from 50 solutions, each perturbed 10,000 times.

Using the same approach, we found that the accessibility of dual solutions involving hysteretic and adaptive dynamics was 7-fold lower compared to biphasic behavior (A_HA_ = 0.0024 compared to A_HB_ = 0.0170) (Figure 3*A*). This decrease was accompanied by an elevated tension between hysteresis and adaptation (T_HA_ = 0.48 compared to T_HB_ = 0.34). Compared to hysteresis, adaptation is associated with parameters defining moderately enhanced sensor and R modules, and a drastically stronger NFB module (Figure 3*B* and Figure S3 *A*). Changes in parameters associated with PFB were without coherent bias (Text S1, Discussion). Consistent with these results, the dominant shift in KL divergence involved NFB parameters (Figure S3 *B*). Furthermore, the tension (average KL divergence) between hysteretic and biphasic dynamics (0.08) is lower than that between hysteretic and adaptive dynamics (0.11). These observations suggest that an effective strategy to reconcile the drastic tension is to dynamically configure these modules, particularly for the NFB: Increasing its strength favors adaptation, while decreasing it favors hysteresis (Figure 3*C*). Reflecting their ‘hybrid’ nature, dual solutions were concentrated in the overlap between individual dynamics when plotted as a function of sensor and NFB strengths (Figure S3 *A* and *C*).

To examine the specific contribution of NFB and sensor in modulating these dynamics, we varied its strength in a typical dual solution. Simulations confirmed its ability to generate both dynamics (Figure 3*D*). Weakening the NFB module (by substituting it with the median value from hysteresis solutions) eliminated the adaptive response and strengthening it (by substituting it with the median value from adaptive solutions) increased the precision of adaptation, consistent with its requirement for this behavior [25]. Weakening the NFB module also enhanced hysteresis to the point that E2F expression became irreversible (i.e. the solid and dotted curves do not meet at low serum). Yet, strengthening it diminished hysteresis by interfering with maintenance of the E2F ON state upon reduction in serum. The sensor strength had a more general impact (Figure S3 *D*). The sensor strength for the dual solution seemed to be near optimal for hysteresis; either weakening or strengthening it led to almost elimination of hysteresis.

The strong tension between dynamics corresponds to a greatly reduced dual accessibility and suggests that they may be operational over a much restricted parameter space (Figure 3*C*). Indeed, here tension penalized the performance of individual dual solutions: Dual solutions were significantly less resilient to perturbations than single solutions in maintaining both hysteretic and adaptive dynamics (Figure 3*E* and Figure S3 *E*).

The drastically reduced accessibility and robustness of dual solutions suggests that they would be ineffective in accommodating both dynamics. Instead, dynamic network reconfiguration is likely critical, which is consistent with the operation of the network: the negative feedback on E2F has a significant time-delay in its operation. In the G_1_ phase, the Anaphase-Promoting Complex/Cdh1 (APC^Cdh1^) keeps negative feedback from both CYC_A_ [26] and SKP2 [27] low by targeting them for proteasomal-mediated degradation. Upon progression to G_1_/S, E2F activity increases and induces CYC_E_ - which is resistant to APC^Cdh1^ - engaging sole positive feedback. SKP2 and CYC_A_ levels are eventually allowed to increase through E2F-mediated induction of Emi1 [28] which targets APC^Cdh1^ for destruction. This is reinforced through positive feedback as CYC_A_ itself can also target APC^Cdh1^ for destruction [29]. Delay is also achieved at the transcriptional level through ordered release of *Cyclin E* and *Cyclin A* from RB-mediated repression [30]. This temporal coordination has been speculated to enforce a brief time window between DNA replication origin licensing mediated by CYC_E_ and origin deactivation and initiation of DNA synthesis mediated by CYC_A_ [31], [32]. Sequential triggering of positive and negative feedback appears to be a generic, systems-level organizational principle of networks underlying cell cycle control conserved throughout evolution [33], [34]. Our analysis suggests an additional role for the temporal coordination: it represents dynamic network reconfiguration that accommodates robust hysteretic and adaptive E2F responses.

**Figure 4:**
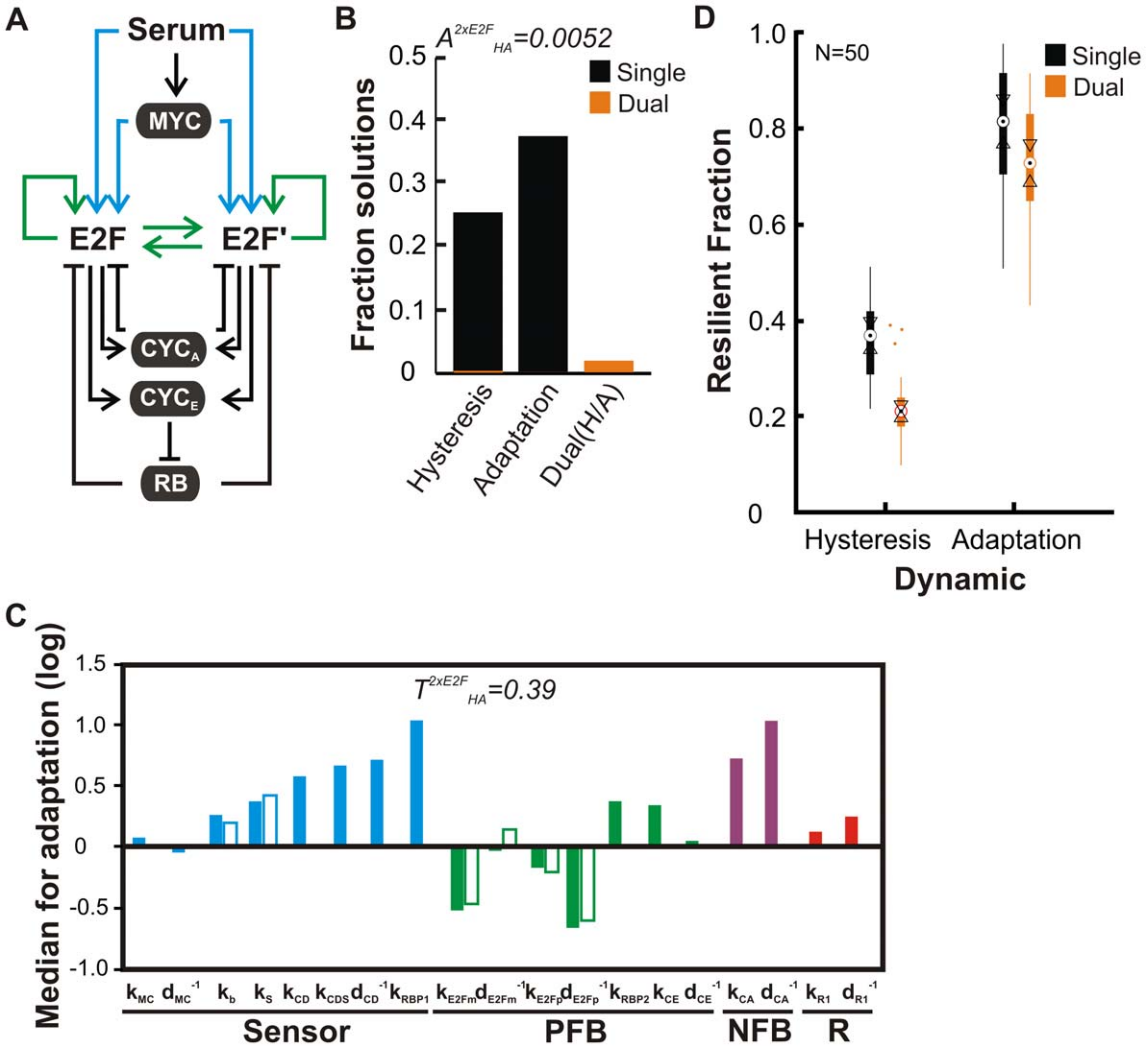
Network complexity mitigates tension. *(A)* Extended model includes duplicated E2F species (E2F′) with independent regulation of synthesis and degradation. E2F dynamics are the sum of E2F and E2F′. All other interactions described in Figure 1*B* are maintained but omitted here for clarity. *(B)* Fraction of parameter sets resulting from 10,000 algorithm iterations that support hysteresis (n = 2,508), adaptation (n = 3,704) or dual dynamics (n = 182). *(C)* Network configuration for adaptation relative to hysteresis. Parameters specific to E2F′ are shown by open bars and all others are shared between the two E2F species. *(D)* Resilience of solutions in the vicinity of the median for hysteresis, adaptation, and Dual(H/A) data. Each boxplot summarize the results from 50 solutions, each perturbed 10,000 times.

In addition, the tension between dynamics can potentially be alleviated by increasing network complexity. For example, eight E2F members of the E2F family have been identified in mammals; some members can functionally substitute for one another [35]. E2F1 and E2F3 are part of the “activator” subgroup required for cell cycle entry of fibroblasts from quiescence [36]. We wondered if such apparent redundancy could reduce tension. To test this notion, we extended our model to include an additional E2F member (E2F′) expressed in parallel with E2F (Figure 4*A* and Text S1, Mathematical Model). In particular, the model includes distinct parameters governing production and degradation of each E2F copy. On the other hand, we assumed that the biochemical activity of each E2F copy was indistinguishable and could contribute in an additive manner to overall E2F output (i.e. hysteresis and adaptation) as well as to downstream gene expression (i.e. Cyclin E and Cyclin A) via shared parameters.

The added complexity indeed led to a 3.1-fold increase in dual solution accessibility (A^2xE2F^_HA_ = 0.0052 compared to A_HA_ = 0.0024) (Figure 4*B*). This was accompanied by a reduction in network tension with dual E2F (T^2xE2F^_HA_ = 0.39 versus T_HA_ = 0.48) (Figure 4*C*). This is reflected in the more modest extent to which the NFB and R modules shifted between hysteretic and adaptive dynamics (Figure 4*C* and Figure S4 *A*) and the greater extent of overlap in their distributions (Figure S4 *B*). Importantly, inclusion of an additional E2F copy was sufficient to increase the resilience of dual solution adaptation such that the median was not significantly different from single task solutions (Figure 4*D*). In contrast, this additional complexity did not have a significant impact on the resilience of hysteresis associated with dual solutions. Why this fragility of hysteretic dynamics persists in such dual solutions is not clear. Nevertheless, these results are consistent with the notion that increasing network complexity reduces tension and the corresponding penalty on some aspects of robustness.

## Discussion

**Figure 5:**
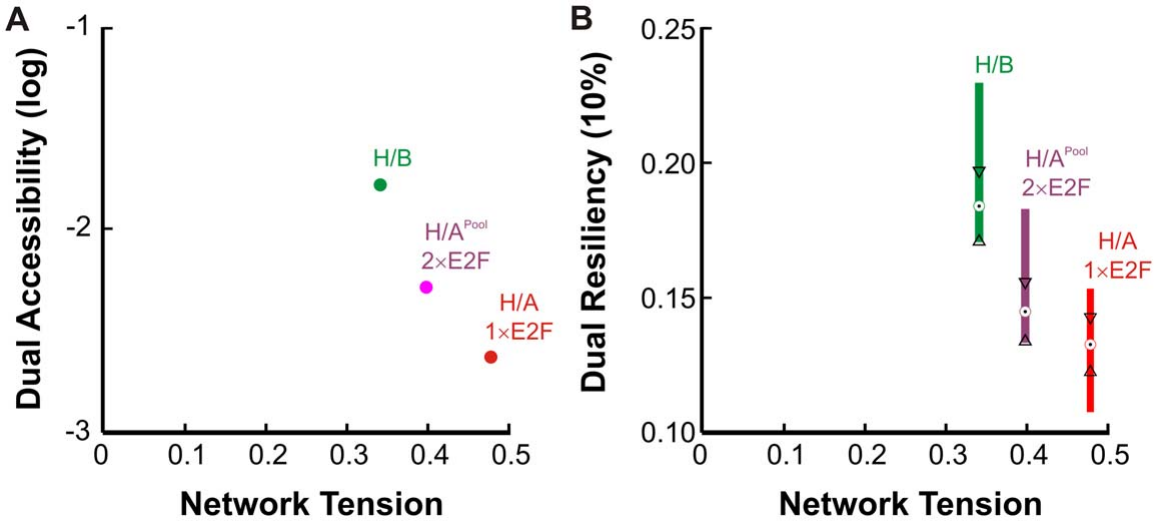
Correlation between tension and robustness. *(A)* Relationship between tension and the accessibility of dual solutions for each pair of dynamics examined. Abbreviations: H/B – hysteresis paired with biphasic; H/A – hysteresis paired with adaptation; 1×E2F – single E2F model; 2×E2F – double E2F model. *(B)* Relationship between tension and resiliency to parameter perturbation. Boxplots span inter-quartile range and medians are indicated with a circle for 50 dual solutions in each category. Two medians are significantly different at the 5% level if the interval between triangular notches do not overlap. Data taken from results presented in Figure 2,3, and 4.

Quantitative modeling has been widely adopted to examine design principles of biological networks. Many studies have provided important insight into the ways networks generate particular dynamic responses [25], [37]. To date, however, how a multitasking cellular network accommodates different dynamics is poorly understood, despite the recognition of their wide presence and importance. Here we have developed a general approach to quantify tension between different dynamics, which we have applied to a well-established network underlying cell cycle progression. In general, our analysis is consistent with an inverse relationship between tension and the overall robustness of network operation (Figure 5). In the face of moderate tension, common or ‘one-size-fits-all’ parameter sets could be attractive as they avoid the need for additional, possibly complex, mechanisms to coordinate system parameters. However, dynamic network reconfiguration may be critical to resolve strong tension. Though dual solutions exist, there is a pronounced penalty on the accessibility and resilience of these solutions. Our approach is general in that it can be applied to any other network with behaviors that are distinct and quantifiable. In the case where a network demonstrates numerous tasks (including the RB-E2F network), accessibility, tension, and resiliency can be reported by an “adjacency matrix”, reporting all interactions in a pair-wise fashion.

Our findings have several implications for our understanding of the RB-E2F switch as well as a variety of other multitasking networks (Table 1). First, our generic framework provides additional criteria to assess model selection, sometimes favoring choices that are not intuitive. In the case of the RB-E2F network, the relatively high tension between hysteretic and adaptive tasks suggests a critical need for additional mechanisms able to delay negative feedback (i.e. CYC_A_) or buffer parameter changes (i.e. E2F duplication). Another example involves a study by Ashall *et al.* [38] concerning how different pulsatile TNF-α input patterns encode unique NF-κB nuclear translocation dynamics (Figure S5 *A*). The authors were prompted to propose an alternative network model wiring when they were unable to find common parameter sets that could satisfy all NF-κB tasks using a traditional model. Using their data, we calculated that the tension between two tasks (“Continuous” and “60 minute”) was reduced from T_Con/60_ = 1.69 to T′_Con/60_ = 0.84 in the alternative model along with a corresponding increase in accessibility from A_Con/60_ = 0 to A′_Cont/60_ = 0.29 (Figure S5 *B*). What system-wide values of tension and accessibility are across all model parameters remains to be seen. Nonetheless, our study suggests that dynamic shifting of parameters is more desirable from the perspective of robustness.

**Table 1:** Examples of tension and coordination in biological networks.

Network	Stimulus	Tasks	Module	Reference
RB-E2F	Serum, MYC	Bistable; Biphasic	Sensor, R	This study; [30], [32]
	Serum	Bistable; Adaptive	Sensor, NFB	
NF-κB	TNF-α	“Continuous”; “60 min”;“100 min”;“200 min”	IKK feedback	[38], [49], [50]
Notch/Delta	Achete/Schute	“2 cell”;“7 cell”;“Line”	?	[39]
p53	γ-radiation	Digital pulses	NFB	[4]
	UV	Analogue pulse		
MAPK	EGF	Transient, graded	NFB	[42], [51]
	NGF	Sustained, bistable	PFB	

Second, tension has the potential to affect network evolvability [6]. In particular, coopting additional functions could interfere with pre-existing network dynamics (i.e. partial overlap of solution space), thereby reducing the ability of the network to tolerate additional alterations. For example, Meir *et al*. [39] modeled the ability of the Notch-Delta signaling network [40] to generate three spatial cell fate patterns – “2-cell”, “7-cell” and “Line” –attributed to the pathway during animal development. They showed that the solution spaces for these tasks were only partially overlapping (Figure S5 *C* and *D*): Only 25% of solutions for the “2-cell” tasks could accommodate a “7-cell” pattern while nearly 80% of “7-cell” solutions could also produce “2-cell” patterning corresponding to an accessibility of A_2–7_ = 0.51 for dual solutions. Also, parameters for “Line” overlap to an even lesser extent with solutions for the two other tasks. From this, the authors speculated that existence of universal parameter sets represent an evolutionarily feasible route towards the goal of achieving novel functions. On the other hand, these same observations offer direct support for our argument that tension *reduces* robustness and constrains a network's capacity to adopt additional tasks. An intriguing possibility is that dynamic shifting, increased complexity, or other strategies may enable a network such as this to increase its workload [41].

Third, by focusing on the coordination of different tasks, our methodology can provide novel, experimentally testable hypotheses concerning what mechanisms are tied to potential conflicts between dynamics and how they are resolved. For example, Santos *et al.* [42] showed that the MAPK cascade, consisting of RAF, MEK1/2, and ERK1/2, demonstrates distinct dynamics and contrasting phenotypes in response to EGF and NGF (Figure 1 and Figure S6 *A*). Importantly, EGF stimulated negative feedback between ERK and RAF whereas NGF stimulated positive feedback. The growth-factor context-dependent MAPK topologies are a clear example of tension between dynamics and the functional role of network reconfiguration. Analogous to our results showing that modulating NFB strength could impact hysteresis (Figure 3*D*), small-molecules used to constitutively suppress and sustain positive feedback could swap ERK dynamics and physiological effects of NGF and EGF. Furthermore, the authors showed that partial activation of positive feedback via interfering RNA (RNAi) generated an intermediate ability of EGF to induce differentiation, suggesting a quantitative relationship between tension and phenotypic outcome.

Another example involves the multifunctional response of the p53 tumor suppressor. Batchelor et al. [4] demonstrated that repeated, digital pulses stimulated by γ-radiation (γ-IR) required WIP1-mediated negative feedback whereas UV radiation generated a single, graded p53 response (Figure S6 *B*). Importantly, suppression of negative feedback by RNAi against WIP1 was sufficient for γ-IR to generate a p53 response characteristic of UV [43]. These observations represent a clear demonstration of tension between dynamics attributed to negative feedback, and its reconciliation through duplication and diversification of the network (i.e. ATM and ATR). In retrospect, our framework provides a rational means to identify such network tension, which may not easily arise from intuition alone or even a deep knowledge of the network, especially when tension arises from subtle and/or multiple parameter shifts.

For the RB-E2F network, our detailed examination of tension and robustness provide experimentally testable hypotheses. First, our results suggest that the strength of negative feedback acting upon E2F is inversely related to the extent of hysteresis. The strength and timing of NFB could be realized through a small-molecule inducible Cyclin A expression construct. Alternatively, premature Cyclin A activity could be achieved through introduction of an N-terminal deletion mutant resistant to APC/C-mediated destruction [44]. The effect of this on the E2F dose-response to serum could be readily achieved using a previously devised fluorescent reporter for *E2f1* [45]. Second, this same experimental system could be used to test the hypothesis that additional copies of E2F insulate the hysteretic response from premature or intensified NFB. Finally, our results lead directly to the hypothesis that strong NFB will reduce the robustness of networks able to accommodate both dynamics. Such a question would be best suited using a synthetic biology approach and predicts that circuits with both bistable and adaptive dynamics would arise with relatively mild NFB.

If tension places a fundamental constraint on the operation and architecture of multifunctional networks, it has implications for engineering of synthetic biological systems. To date, most efforts have focused on engineering of gene circuits with limited, dedicated functions. More complex functions can then be realized by integrating well-defined modules [46], [47]. For those functioning in individual cells, however, this strategy is limited by the ability to insulate different modules as well as the inevitable burden they impose upon cells which can undermine desired functionality [48]. As such, it may be more effective to explore strategies that include dynamic network reconfiguration to perform multiple functions in a robust manner. In this case, synthetic biology may take a cue from nature: Rather than attempting to generate an ever-expanding toolkit of biological components, an emphasis will be placed back upon the vast potential in differential regulation of existing entities.

## Methods

### Numerical simulations

Simulations were performed with Matlab, version R12 (Mathworks, Natick MA) employing the ode15 solver.

## Supporting Information

Figure S1 **Objective functions for search algorithm and convergence.** (*A*) Objective functions used to quantify numerical simulation output. (*Left*) Hysteresis is defined as a minimal path difference (ΔP = 0.5) in E2Fm at 24 hours after an increase in serum from 0.01% or decreasing from 10%. This was calculated by applying the Matlab function *trapz* to the difference in steady-state E2F values generated by decreasing and increasing serum. (*Center*) The relative adaptation in E2Fp was calculated by ΔF/ΔI over 25 hours and a minimum threshold of 0.80 defines a solution. ΔI is the difference between initial and peak levels and a minimal ΔI is enforced to filter out trivial solutions. ΔF is the difference between peak and final levels. (*Right*) Biphasic behavior is defined by the extent of E2Fm suppression relative to initial increase (ΔF/ΔI) at 36 hours after a change in MYC synthesis rate (parameter ke_MYC_). A minimum threshold of ΔF/ΔI = 0.80 defines a solution. A minimal absolute value of ΔI also applies in this case. It should be noted that hysteresis, adaptation, and biphasic behavior could be measured at the protein level without loss of generality. (*B*) (*Left*) Fraction of algorithm iterations that lead to identification of a solution for different total numbers of algorithm iterations. (*Right*) Calculation of dual accessibility for different number of total algorithm iterations. Data is a subset of data presented on left.
(TIF)

Figure S2 **Raw data for solutions to hysteresis and biphasic responses.** (*A*) Distribution of solution parameters supporting hysteresis, biphasic dose-response, dual tasks (Dual(H/B)^Product^), dual tasks with initial parameters that were solutions of single tasks (Dual(H/B)^Single task IC^), and dual tasks using an objective composed of the sum of individual objectives (Dual(H/B)^Additive^). Boxplots summarize distribution of values (logarithm) for solution parameters. Medians are indicated by circles; lower and upper end of boxes are 1st and 3rd quartiles, respectively; Medians are significantly different at the 5% level if interval between triangular notches are non-overlapping. Whiskers span region 1.5 times the inter-quartile range; individual points outside of this are shown and perturbed from the center for clarity. Parameters are expressed such that value increases with strength of module. Abbreviations: PFB –positive feedback; NFB – negative feedback; R- repression. (B) Kullback-Leibler (KL) distance for solution parameters of hysteretic and biphasic tasks. Tension is the average distance over parameters. (*C*) Module strength for solutions. Value along each axis is the logarithm of the product over all module parameters. (*D*) Numerical simulations. The Dual(H/B) solution is the same used in Figure 2*D*. Sensor strength was decreased and increased by substituting median value for hysteresis (−Sensor) and biphasic (+Sensor), respectively. The value of d_E2Fp_^−1^ was increased (+d_E2Fp_^−1^) by using the median value from hysteresis. (*E*) Evaluation of resilience for representative solutions. The change in objective score relative to original is plotted as a function of total parameter variation (K). Shown are results of 10,000 perturbations. Resilience of a perturbed parameter set is that maintaining at least 10% of its score (i.e. log value greater than −1).
(TIF)

Figure S3 **Raw data for solutions to hysteresis and adaptation.** (*A*) Distribution of solution parameters values for hysteretic and adaptive responses to serum. Boxplots summarize the distribution of values (logarithm) of solution parameters. See legend for Figure S2 *A* for details. (B) KL divergence for solution parameters of hysteretic and adaptive tasks. (*C*) Module strength for solutions to each dynamic. Module strength on each axis is the logarithm of the product over all module parameters. (*D*) Numerical simulations of the same Dual(H/A) solution as described in Figure 3*D*. Sensor strength was decreased and increased by substituting median value from hysteresis (−Sensor) and adaptation (+Sensor), respectively. The value of k_E2Fm_ and d_E2Fp_^−1^ were increased (+k_E2Fm_,d_E2Fp_^−1^) by using the median value from hysteresis. (*E*) Evaluation of resilience for representative solutions. See legend for Figure S2 *E* for detailed description.
(TIF)

Figure S4 **Raw data for solutions to hysteresis and adaptation for model with duplicated E2F.** (*A*) Distribution of solution parameters values for hysteretic and adaptive responses to serum. (*B*) Module strength for solutions to each dynamic.
(TIF)

Figure S5 **Multifunctional networks with diverse, context-specific dynamics.** (*A* and *B*) Tension in the NF-κB multitasking network. (*A*) The NF-κB pathway mediates stress signals including those from the TNF-α cytokine. Cells treated with different temporal patterns of TNF-α given in 5 minute pulses display distinct NF-κB dynamic ‘tasks’. (*B*) A previous ‘traditional’ model of the pathway is unable to accommodate all tasks with a common parameter set whereas an alternative, “Triple-feedback” model with Iκκ feedback is able to. Tension and accessibility of dual solutions involving the continuous and 60 minute TNF-α pulsing protocols were calculated from data using the A20 degradation rate parameter. Adapted from Ashall et al. [25]. (*C* and *D*) Tension in the Notch-Delta multitasking network. (*C*) Notch-Delta signaling leads to differential expression of Achete (AC)/Schute (SC) and binary cell fate patterning in adjacent cells during fruit fly development. The network is able to translate an initial pattern of AC/SC expressed at moderate levels into a final ON/OFF pattern. (*D*) Calculation of accessibility of dual 2- and 7-cell pattern. Orange bars indicate subset of parameters for each single task that are dual. Accessibility calculated from data presented by Meir et al. [26].
(TIF)

Figure S6 **Multifunctional networks with diverse, context-specific dynamics.** (*A*) The MAPK pathway multitasks. Stimulation of neuronal precursor cells with EGF and NGF elicit distinct dynamics and translate into opposite phenotypic outcomes. Protein Kinase C (PKC) is required but not sufficient for positive feedback. Small molecules used to sustain positive feedback (phorbol-12-myristate-13-acetate (PMA)) or preclude it (Go7874) were sufficient to swap EGF- and NGF-mediated dynamics and cellular outcomes. Adapted from [27]. (*B*) The p53 stress response pathway multitasks. p53 can mediate cell stress signals and controls the expression of genes that mitigate their effects. Double-strand (ds) breaks induced by ionizing radiation induce recurrent pulses of p53 that are whose amplitude is dose-independent [28]; Single-strand (ss) DNA adducts induced by UV cause a large pulse of p53 that is graded in terms of peak response [29]. Colored links indicate interactions (i.e., synthesis rate in blue and NFB in red) activated in a stimulus-specific fashion. The p53 network has also been shown to respond to a large panel of cell stresses and other physiological contexts, with dynamics that are poorly understood.
(TIF)

Text S1 **Detailed description of modeling and mathematical framework.** The supporting text opens with a discussion section describing the theoretical model of the RB-E2F switch underlying mammalian cell cycle control along with a discussion of the role of positive feedback module in the adaptive and biphasic E2F responses. Following this is a materials and methods section that describes the computational approach used to identify parameter solutions that satisfy RB-E2F network dynamics. Concluding this material are definitions of tension, Kullback-Leibler divergence, and measures of robustness.
(DOC)

## References

[1] Marshall CJ (1995). Specificity of receptor tyrosine kinase signaling: transient versus sustained extracellular signal-regulated kinase activation.. Cell.

[2] Vogelstein B, Lane D, Levine AJ (2000). Surfing the p53 network.. Nature.

[3] Lahav G, Rosenfeld N, Sigal A, Geva-Zatorsky N, Levine AJ (2004). Dynamics of the p53-Mdm2 feedback loop in individual cells.. Nat Genet.

[4] Batchelor E, Loewer A, Mock C, Lahav G (2011). Stimulus-dependent dynamics of p53 in single cells.. Mol Syst Biol.

[5] Batchelor E, Loewer A, Lahav G (2009). The ups and downs of p53: understanding protein dynamics in single cells.. Nat Rev Cancer.

[6] Kirschner M, Gerhart J (1998). Evolvability.. Proc Natl Acad Sci U S A.

[7] Arora JS, Marler RT (2004). Survey of multi-objective optimization methods for engineering.. Struct Multidisc Optim.

[8] Handl J, Kell DB, Knowles J (2007). Multiobjective optimization in bioinformatics and computational biology.. IEEE/ACM Trans Comput Biol Bioinform.

[9] Harbour JW, Dean DC (2000). The Rb/E2F pathway: expanding roles and emerging paradigms.. Genes Dev.

[10] Nevins JR (2001). The Rb/E2F pathway and cancer.. Hum Mol Genet.

[11] Leung JY, Ehmann GL, Giangrande PH, Nevins JR (2008). A role for Myc in facilitating transcription activation by E2F1.. Oncogene.

[12] Johnson DG, Ohtani K, Nevins JR (1994). Autoregulatory control of E2F1 expression in response to positive and negative regulators of cell cycle progression.. Genes Dev.

[13] Ohtani K, DeGregori J, Nevins JR (1995). Regulation of the cyclin E gene by transcription factor E2F1.. Proc Natl Acad Sci U S A.

[14] Krek W, Ewen ME, Shirodkar S, Arany Z, Kaelin WG (1994). Negative regulation of the growth-promoting transcription factor E2F-1 by a stably bound cyclin A-dependent protein kinase.. Cell.

[15] Marti A, Wirbelauer C, Scheffner M, Krek W (1999). Interaction between ubiquitin-protein ligase SCFSKP2 and E2F-1 underlies the regulation of E2F-1 degradation.. Nat Cell Biol.

[16] O'Donnell KA, Wentzel EA, Zeller KI, Dang CV, Mendell JT (2005). c-Myc-regulated microRNAs modulate E2F1 expression.. Nature.

[17] Martelli F, Hamilton T, Silver DP, Sharpless NE, Bardeesy N (2001). p19ARF targets certain E2F species for degradation.. Proc Natl Acad Sci U S A.

[18] Yao G, Lee TJ, Mori S, Nevins JR, You L (2008). A bistable Rb-E2F switch underlies the restriction point.. Nat Cell Biol.

[19] Pardee AB (1974). A restriction point for control of normal animal cell proliferation.. Proc Natl Acad Sci U S A.

[20] Wong JV, Yao G, Nevins JR, You L (2011). Viral-Mediated Noisy Gene Expression Reveals Biphasic E2f1 Response to MYC.. Mol Cell.

[21] Leone G, DeGregori J, Yan Z, Jakoi L, Ishida S (1998). E2F3 activity is regulated during the cell cycle and is required for the induction of S phase.. Genes Dev.

[22] Timmers C, Sharma N, Opavsky R, Maiti B, Wu L (2007). E2f1, E2f2, and E2f3 control E2F target expression and cellular proliferation via a p53-dependent negative feedback loop.. Mol Cell Biol.

[23] Pickering MT, Stadler BM, Kowalik TF (2008). miR-17 and miR-20a temper an E2F1-induced G1 checkpoint to regulate cell cycle progression.. Oncogene.

[24] Krek W, Xu G, Livingston DM (1995). Cyclin A-kinase regulation of E2F-1 DNA binding function underlies suppression of an S phase checkpoint.. Cell.

[25] Ma W, Trusina A, El-Samad H, Lim WA, Tang C (2009). Defining network topologies that can achieve biochemical adaptation.. Cell.

[26] Peters JM (2002). The anaphase-promoting complex: proteolysis in mitosis and beyond.. Mol Cell.

[27] Wei W, Ayad NG, Wan Y, Zhang GJ, Kirschner MW (2004). Degradation of the SCF component Skp2 in cell-cycle phase G1 by the anaphase-promoting complex.. Nature.

[28] Machida YJ, Dutta A (2007). The APC/C inhibitor, Emi1, is essential for prevention of rereplication.. Genes Dev.

[29] Sorensen CS, Lukas C, Kramer ER, Peters JM, Bartek J (2001). A conserved cyclin-binding domain determines functional interplay between anaphase-promoting complex-Cdh1 and cyclin A-Cdk2 during cell cycle progression.. Mol Cell Biol.

[30] Zhang HS, Gavin M, Dahiya A, Postigo AA, Ma D (2000). Exit from G1 and S phase of the cell cycle is regulated by repressor complexes containing HDAC-Rb-hSWI/SNF and Rb-hSWI/SNF.. Cell.

[31] Coverley D, Laman H, Laskey RA (2002). Distinct roles for cyclins E and A during DNA replication complex assembly and activation.. Nat Cell Biol.

[32] Mailand N, Diffley JF (2005). CDKs promote DNA replication origin licensing in human cells by protecting Cdc6 from APC/C-dependent proteolysis.. Cell.

[33] Eser U, Falleur-Fettig M, Johnson A, Skotheim Jan M (2011). Commitment to a Cellular Transition Precedes Genome-wide Transcriptional Change.. Molecular Cell.

[34] Ferrell James E (2011). Simple Rules for Complex Processes: New Lessons from the Budding Yeast Cell Cycle.. Molecular Cell.

[35] Tsai SY, Opavsky R, Sharma N, Wu L, Naidu S (2008). Mouse development with a single E2F activator.. Nature.

[36] Wu L, Timmers C, Maiti B, Saavedra HI, Sang L (2001). The E2F1-3 transcription factors are essential for cellular proliferation.. Nature.

[37] Tsai TY, Choi YS, Ma W, Pomerening JR, Tang C (2008). Robust, tunable biological oscillations from interlinked positive and negative feedback loops.. Science.

[38] Ashall L, Horton CA, Nelson DE, Paszek P, Harper CV (2009). Pulsatile stimulation determines timing and specificity of NF-kappaB-dependent transcription.. Science.

[39] Meir E, von Dassow G, Munro E, Odell GM (2002). Robustness, flexibility, and the role of lateral inhibition in the neurogenic network.. Curr Biol.

[40] Artavanis-Tsakonas S, Rand MD, Lake RJ (1999). Notch signaling: cell fate control and signal integration in development.. Science.

[41] Duboule D, Wilkins AS (1998). The evolution of ‘bricolage’.. Trends Genet.

[42] Santos SD, Verveer PJ, Bastiaens PI (2007). Growth factor-induced MAPK network topology shapes Erk response determining PC-12 cell fate.. Nat Cell Biol.

[43] Batchelor E, Mock CS, Bhan I, Loewer A, Lahav G (2008). Recurrent initiation: a mechanism for triggering p53 pulses in response to DNA damage.. Mol Cell.

[44] Geley S, Kramer E, Gieffers C, Gannon J, Peters JM (2001). Anaphase-promoting complex/cyclosome-dependent proteolysis of human cyclin A starts at the beginning of mitosis and is not subject to the spindle assembly checkpoint.. J Cell Biol.

[45] Yao G, Tan C, West M, Nevins JR, You L (2011). Origin of bistability underlying mammalian cell cycle entry.. Mol Syst Biol.

[46] Nandagopal N, Elowitz MB (2011). Synthetic biology: integrated gene circuits.. Science.

[47] Purnick PE, Weiss R (2009). The second wave of synthetic biology: from modules to systems.. Nat Rev Mol Cell Biol.

[48] Tan C, Marguet P, You L (2009). Emergent bistability by a growth-modulating positive feedback circuit.. Nat Chem Biol.

[49] Werner SL, Barken D, Hoffmann A (2005). Stimulus specificity of gene expression programs determined by temporal control of IKK activity.. Science.

[50] Covert MW, Leung TH, Gaston JE, Baltimore D (2005). Achieving stability of lipopolysaccharide-induced NF-kappaB activation.. Science.

[51] Sasagawa S, Ozaki Y, Fujita K, Kuroda S (2005). Prediction and validation of the distinct dynamics of transient and sustained ERK activation.. Nat Cell Biol.

