# Supplementary figures and images for: Tension and Robustness in Multitasking Cellular Networks
Source: PLoS Comput Biol. 2012 Apr 26;8(4):e1002491. doi: 10.1371/journal.pcbi.1002491 (PMC3343128; doi:10.1371/journal.pcbi.1002491)

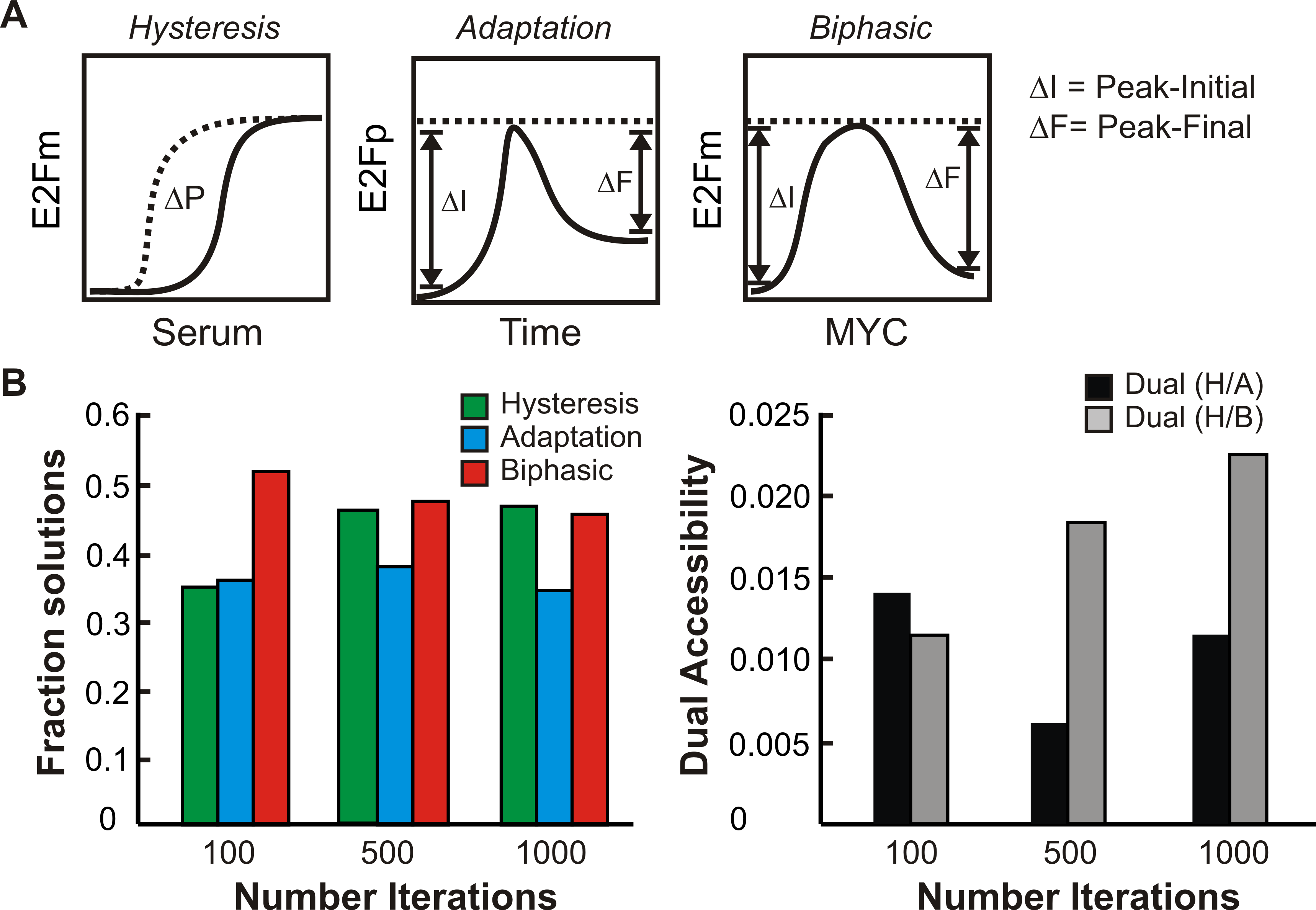

Supplement: Figure S1 — Objective functions for search algorithm and convergence. (A) Objective functions used to quantify numerical simulation output. (Left) Hysteresis is defined as a minimal path difference (ΔP = 0.5) in E2Fm at 24 hours after an increase in serum from 0.01% or decreasing from 10%. This was calculated by applying the Matlab function trapz to the difference in steady-state E2F values generated by decreasing and increasing serum. (Center) The relative adaptation in E2Fp was calculated by ΔF/ΔI over 25 hours and a minimum threshold of 0.80 defines a solution. ΔI is the difference between initial and peak levels and a minimal ΔI is enforced to filter out trivial solutions. ΔF is the difference between peak and final levels. (Right) Biphasic behavior is defined by the extent of E2Fm suppression relative to initial increase (ΔF/ΔI) at 36 hours after a change in MYC synthesis rate (parameter keMYC). A minimum threshold of ΔF/ΔI = 0.80 defines a solution. A minimal absolute value of ΔI also applies in this case. It should be noted that hysteresis, adaptation, and biphasic behavior could be measured at the protein level without loss of generality. (B) (Left) Fraction of algorithm iterations that lead to identification of a solution for different total numbers of algorithm iterations. (Right) Calculation of dual accessibility for different number of total algorithm iterations. Data is a subset of data presented on left. (TIF) [file pcbi.1002491.s001.tif]

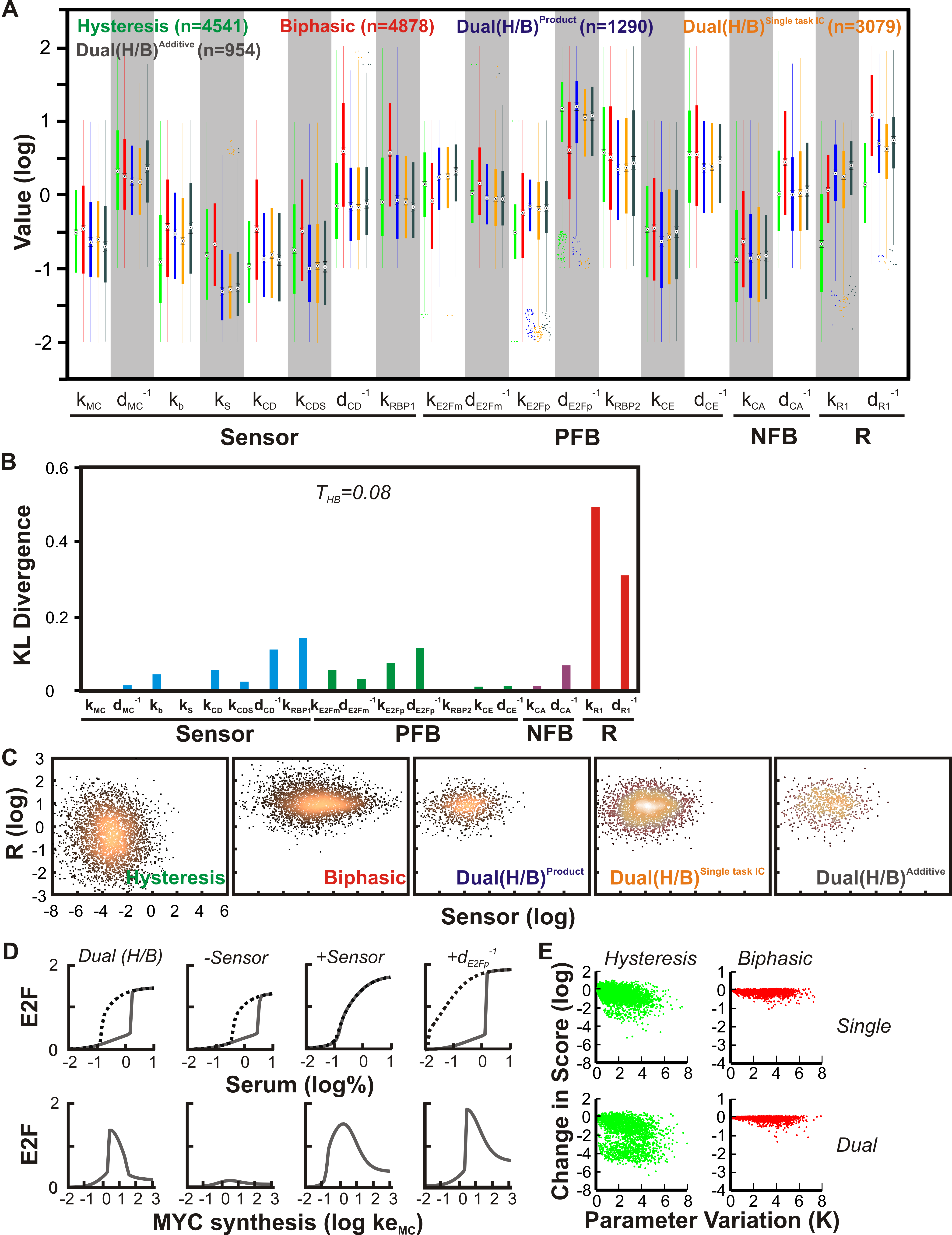

Supplement: Figure S2 — Raw data for solutions to hysteresis and biphasic responses. (A) Distribution of solution parameters supporting hysteresis, biphasic dose-response, dual tasks (Dual(H/B)Product), dual tasks with initial parameters that were solutions of single tasks (Dual(H/B)Single task IC), and dual tasks using an objective composed of the sum of individual objectives (Dual(H/B)Additive). Boxplots summarize distribution of values (logarithm) for solution parameters. Medians are indicated by circles; lower and upper end of boxes are 1st and 3rd quartiles, respectively; Medians are significantly different at the 5% level if interval between triangular notches are non-overlapping. Whiskers span region 1.5 times the inter-quartile range; individual points outside of this are shown and perturbed from the center for clarity. Parameters are expressed such that value increases with strength of module. Abbreviations: PFB –positive feedback; NFB – negative feedback; R- repression. (B) Kullback-Leibler (KL) distance for solution parameters of hysteretic and biphasic tasks. Tension is the average distance over parameters. (C) Module strength for solutions. Value along each axis is the logarithm of the product over all module parameters. (D) Numerical simulations. The Dual(H/B) solution is the same used in Figure 2D . Sensor strength was decreased and increased by substituting median value for hysteresis (−Sensor) and biphasic (+Sensor), respectively. The value of dE2Fp −1 was increased (+dE2Fp −1) by using the median value from hysteresis. (E) Evaluation of resilience for representative solutions. The change in objective score relative to original is plotted as a function of total parameter variation (K). Shown are results of 10,000 perturbations. Resilience of a perturbed parameter set is that maintaining at least 10% of its score (i.e. log value greater than −1). (TIF) [file pcbi.1002491.s002.tif]

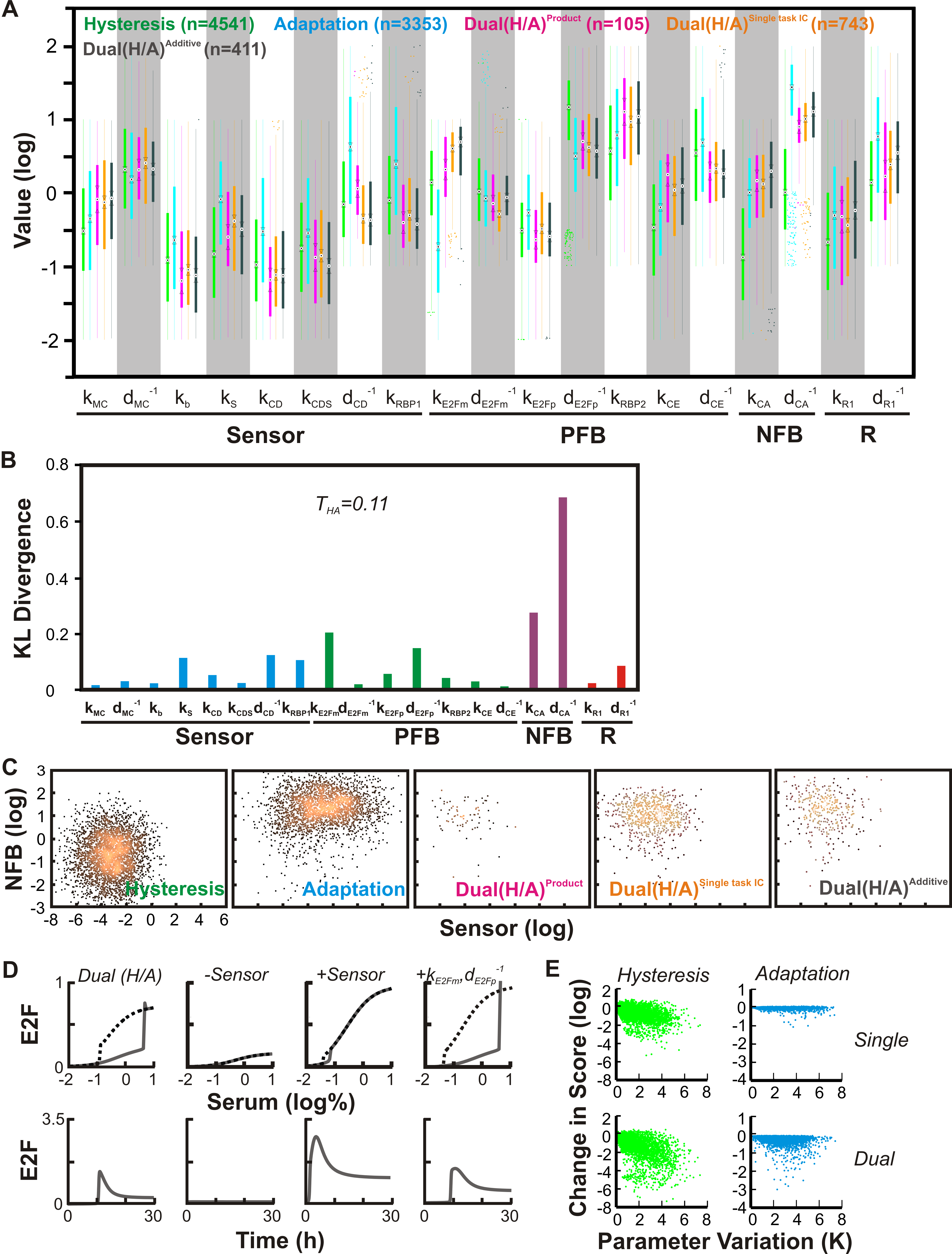

Supplement: Figure S3 — Raw data for solutions to hysteresis and adaptation. (A) Distribution of solution parameters values for hysteretic and adaptive responses to serum. Boxplots summarize the distribution of values (logarithm) of solution parameters. See legend for Figure S2 A for details. (B) KL divergence for solution parameters of hysteretic and adaptive tasks. (C) Module strength for solutions to each dynamic. Module strength on each axis is the logarithm of the product over all module parameters. (D) Numerical simulations of the same Dual(H/A) solution as described in Figure 3D . Sensor strength was decreased and increased by substituting median value from hysteresis (−Sensor) and adaptation (+Sensor), respectively. The value of kE2Fm and dE2Fp −1 were increased (+kE2Fm,dE2Fp −1) by using the median value from hysteresis. (E) Evaluation of resilience for representative solutions. See legend for Figure S2 E for detailed description. (TIF) [file pcbi.1002491.s003.tif]

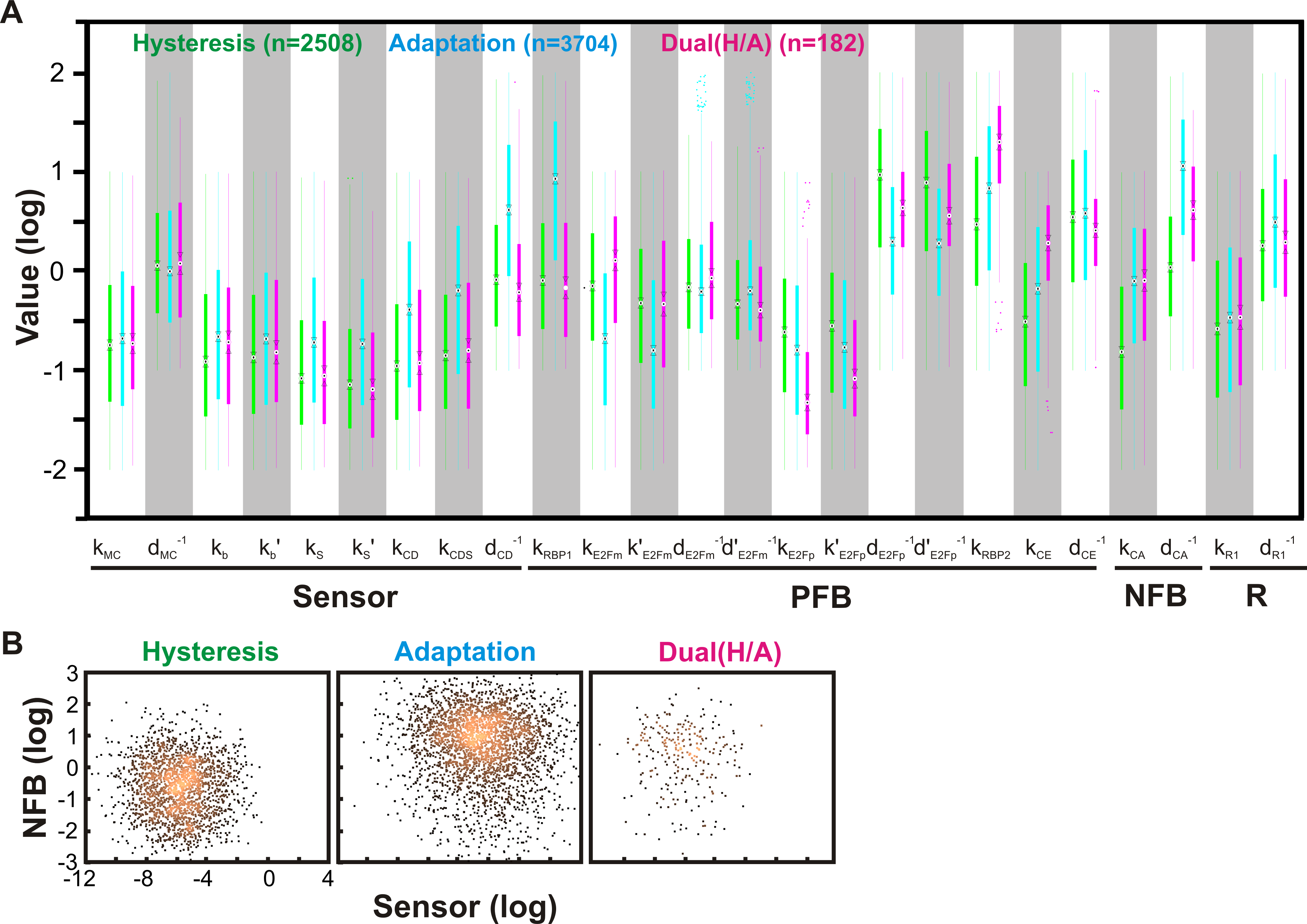

Supplement: Figure S4 — Raw data for solutions to hysteresis and adaptation for model with duplicated E2F. (A) Distribution of solution parameters values for hysteretic and adaptive responses to serum. (B) Module strength for solutions to each dynamic. (TIF) [file pcbi.1002491.s004.tif]

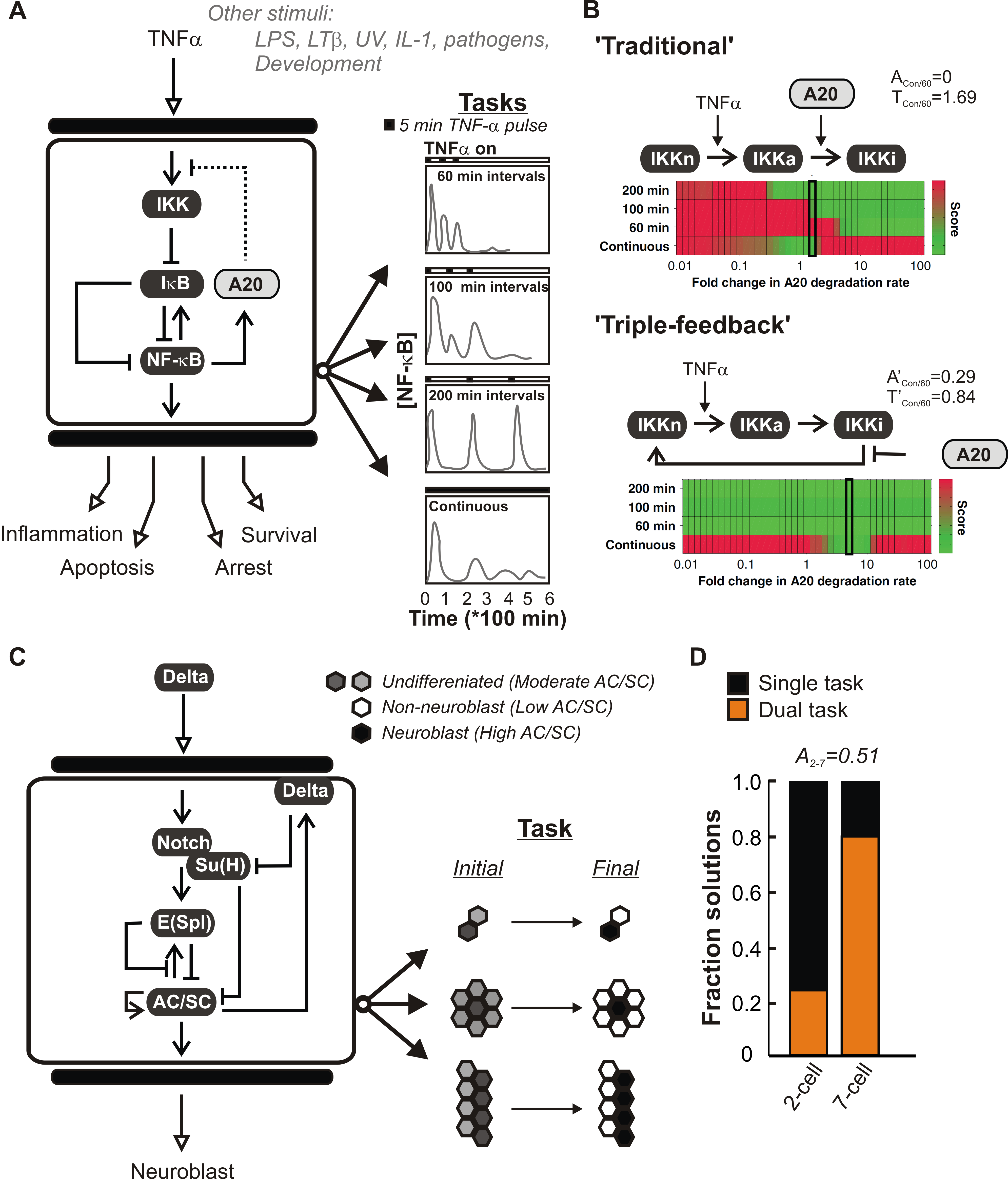

Supplement: Figure S5 — Multifunctional networks with diverse, context-specific dynamics. (A and B) Tension in the NF-κB multitasking network. (A) The NF-κB pathway mediates stress signals including those from the TNF-α cytokine. Cells treated with different temporal patterns of TNF-α given in 5 minute pulses display distinct NF-κB dynamic ‘tasks’. (B) A previous ‘traditional’ model of the pathway is unable to accommodate all tasks with a common parameter set whereas an alternative, “Triple-feedback” model with Iκκ feedback is able to. Tension and accessibility of dual solutions involving the continuous and 60 minute TNF-α pulsing protocols were calculated from data using the A20 degradation rate parameter. Adapted from Ashall et al. [25]. (C and D) Tension in the Notch-Delta multitasking network. (C) Notch-Delta signaling leads to differential expression of Achete (AC)/Schute (SC) and binary cell fate patterning in adjacent cells during fruit fly development. The network is able to translate an initial pattern of AC/SC expressed at moderate levels into a final ON/OFF pattern. (D) Calculation of accessibility of dual 2- and 7-cell pattern. Orange bars indicate subset of parameters for each single task that are dual. Accessibility calculated from data presented by Meir et al. [26]. (TIF) [file pcbi.1002491.s005.tif]

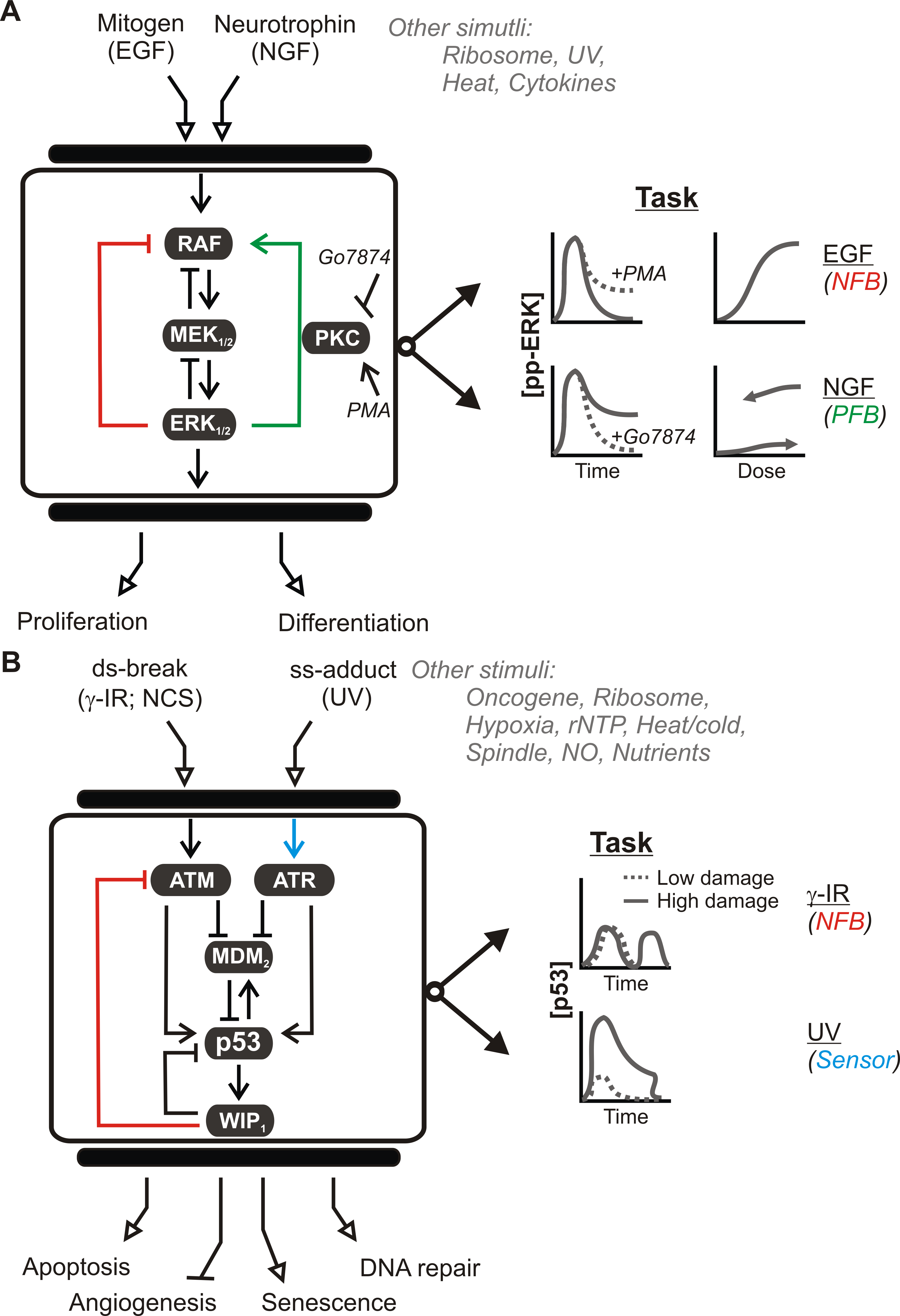

Supplement: Figure S6 — Multifunctional networks with diverse, context-specific dynamics. (A) The MAPK pathway multitasks. Stimulation of neuronal precursor cells with EGF and NGF elicit distinct dynamics and translate into opposite phenotypic outcomes. Protein Kinase C (PKC) is required but not sufficient for positive feedback. Small molecules used to sustain positive feedback (phorbol-12-myristate-13-acetate (PMA)) or preclude it (Go7874) were sufficient to swap EGF- and NGF-mediated dynamics and cellular outcomes. Adapted from [27]. (B) The p53 stress response pathway multitasks. p53 can mediate cell stress signals and controls the expression of genes that mitigate their effects. Double-strand (ds) breaks induced by ionizing radiation induce recurrent pulses of p53 that are whose amplitude is dose-independent [28]; Single-strand (ss) DNA adducts induced by UV cause a large pulse of p53 that is graded in terms of peak response [29]. Colored links indicate interactions (i.e., synthesis rate in blue and NFB in red) activated in a stimulus-specific fashion. The p53 network has also been shown to respond to a large panel of cell stresses and other physiological contexts, with dynamics that are poorly understood. (TIF) [file pcbi.1002491.s006.tif]
