# Supplementary material for: Tension and Robustness in Multitasking Cellular Networks
Source: PLoS Comput Biol. 2012 Apr 26;8(4):e1002491. doi: 10.1371/journal.pcbi.1002491 (PMC3343128; doi:10.1371/journal.pcbi.1002491)
Supplement: Text S1 — Detailed description of modeling and mathematical framework. The supporting text opens with a discussion section describing the theoretical model of the RB-E2F switch underlying mammalian cell cycle control along with a discussion of the role of positive feedback module in the adaptive and biphasic E2F responses. Following this is a materials and methods section that describes the computational approach used to identify parameter solutions that satisfy RB-E2F network dynamics. Concluding this material are definitions of tension, Kullback-Leibler divergence, and measures of robustness. (DOC) [file pcbi.1002491.s007.doc]

**Supporting Text S1**

**Tension and robustness in multitasking cellular networks**

Jeffrey V. Wong^1,2^, Bochong Li^1,2^, and Lingchong You^1,2,3^

^1^Department of Biomedical Engineering, ^2^Institute for Genome Sciences and Policy, and the ^3^Center for Systems Biology, Duke University, Durham, NC, USA, 27708.

Contents

[**1.** **DISCUSSION 3**](#__RefHeading___Toc320206355)

[1.1. Mathematical Model 3](#__RefHeading___Toc320206356)

[• Negative feedback on E2F 3](#__RefHeading___Toc320206357)

[• Repression of E2F 3](#__RefHeading___Toc320206358)

[1.2. Parameter biases in PFB module 4](#__RefHeading___Toc320206359)

[• PFB in adaptation 4](#__RefHeading___Toc320206360)

[• PFB in biphasic response 4](#__RefHeading___Toc320206361)

[2. TABLES 5](#__RefHeading___Toc320206362)

[Table S1. Variable definitions 5](#__RefHeading___Toc320206363)

[Table S2. Equations for single E2F and duplicated E2F models 6](#__RefHeading___Toc320206364)

[Table S3. Model parameters 9](#__RefHeading___Toc320206365)

[3. MATERIALS AND METHODS 11](#__RefHeading___Toc320206366)

[3.1. Parameter search algorithm 11](#__RefHeading___Toc320206367)

[3.2. Definitions 13](#__RefHeading___Toc320206368)

[• Tension 13](#__RefHeading___Toc320206369)

[• Kullback-Leibler divergence 15](#__RefHeading___Toc320206370)

[• Accessibility 15](#__RefHeading___Toc320206371)

[• Resiliency 16](#__RefHeading___Toc320206372)

[4. REFERENCES 17](#__RefHeading___Toc320206373)

# DISCUSSION

# Mathematical Model

## *Negative feedback on E2F*

Negative feedback on E2F has been shown to occur through several possible channels. E2F can activate the expression of *Cyclin A*, which can bind to E2F:DP1 protein complexes and phosphorylate DP1, thereby disrupting the DNA binding capacity of E2F. Likewise, E2F activates transcription of *Skp2* which is a part of the SCF ubiquitin-ligase that in turn targets E2F for proteasomal degradation. E2F is also targeted for proteasomal-mediated degradation through binding to the Arf tumor suppressor , which is a well-studied target of E2F and MYC. An additional source of negative feedback comes from the micro RNA of the miR-17-92 locus. E2F can transcriptionally activate this locus and miRNA in turn target expression of E2F by downregulating transcription. This same locus has also been shown to be regulated by MYC. Here, we lump all of these and possibly other factors that deactivate E2F activity into a single term, CYC_A_ that targets degradation of E2F protein (Table S2).

## *Repression of E2F*

Previous work indicated a biphasic response of E2F to increasing MYC. Our previous analysis of the model (data not shown) implies that an additional source of repression downstream of MYC but upstream of E2F is required to generate this biphasic dose-dependency. Although miRNA and ARF are E2F repressors that are regulated by MYC, we found that their biphasic dynamics were not compatible with biphasic E2F. Thus, we implement E2F repression through (R), which is an unknown intermediate downstream of MYC (Table S2).

# Parameter biases in PFB module

## *PFB in adaptation*

An examination of the parameter distributions for PFB revealed that diminished E2F mRNA synthesis rate (k_E2Fm_) and the increased protein degradation rate (d_E2Fp_) were the only prominent and distinct biases for adaptation (Figure 3*B*). Why might there be a preference for increased E2F protein degradation? It can be shown that the response time of a species with constitutive synthesis is inversely proportional to its half-life. Thus, the increase in d_E2Fp_ (i.e., reduced d_E2Fp_^-1^) might indicate a strong preference for fast E2F protein expression preceding negative feedback. For adaptation, the median value of d_E2Fp_ (~0.32/h) corresponds to a protein half-life on the order of 2 h which may explain why endogenous E2F has such a short half-life ~1-2 h. An alternative explanation for the decrease in k_E2Fm_ and increased d_E2Fp_ is that E2F levels must be restrained in order to allow NFB to correct E2F to near-basal levels. Very high synthesis rates would lead to a high steady-state responses that counteracts resetting of E2F and biologically speaking, proper shut-down of E2F target genes involved in DNA synthesis. This is consistent with numerical simulations showing that increasing the value of k_E2Fm_ and d_E2Fp_^-1^ disturbed the extent of E2F correction (+k_E2Fm_/d_E2Fp_^-1^, Figure S3*D*).

## *PFB in biphasic response*

The only distinct shift in PFB for biphasic behavior was an increase in E2F protein degradation (d_E2Fp_, Figure S2*A*). Akin to the case for adaption, increase in this parameter may aid biphasic behavior by decreasing the steady-state levels of E2F and aid in achieving near-basal levels at high MYC inputs. This is supported by numerical simulations where increasing the value of d_E2Fp_^-1^ (i.e. decreasing d_E2Fp_) perturbed the correction of E2F levels (+d_E2Fp_^-1^, Figure S2*D*).

# TABLES

## Table S1. Variable definitions

| $S$ | Serum and mitogenic stimultion |
| --- | --- |
| $\text{MYC}$ | Product of *c*-*myelocytomastosis* |
| $\text{E2Fm}$ | *E2f* transcription and mRNA |
| $\text{E2F'm}$ | *E2f* duplicate transcription and mRNA |
| $\text{E2Fp}$ | E2F protein |
| $\text{E2F'p}$ | E2F duplicate protein |
| $\text{CA}$ | CYCLIN A |
| $\text{CD}$ | CYCLIN D |
| $\text{CE}$ | CYCLIN E |
| $\text{RB}$ | Pocket proteins p130 and also p107, RB |
| $\text{RE}$ | Complex of RB and E2Fp |
| $\text{RE'}$ | Complex of RB and E2F’p |
| $\text{RP}$ | Phosphorylated RB |
| $\text{R}$ | Repressor |

## Table S2. Equations for single E2F and duplicated E2F models

| **Single E2F model** |
| --- |
| $\frac{\text{d[MYC]}}{\text{dt}}\text{=ke}_{\text{MC}}+\text{k}_{\text{MC}}\cdot\frac{\text{[S]}}{\text{K}_{\text{S}}\text{+[S]}}\text{-d}_{\text{MC}}\cdot\text{[MYC]}$ |
| $\begin{matrix} \frac{\text{d[E2Fm]}}{\text{dt}}\text{=}\left(\text{k}_{\text{S}}\cdot\frac{\text{[S]}}{\text{K}_{\text{S}}\text{+[S]}}\text{+k}_{\text{E2Fm}}\cdot\frac{\text{[MYC]}}{\text{K}_{\text{MC}}\text{+[MYC]}}\cdot\text{ }\frac{\text{[E2Fp]}}{\text{K}_{\text{EF}}\text{+[E2Fp]}}\text{+k}_{\text{b}}\cdot\frac{\text{[MYC]}}{\text{K}_{\text{MC1}}\text{+[MYC]}}\right) \\ \cdot\left(\frac{\text{K}_{\text{R}}}{\text{K}_{\text{R}}\text{+[R]}}\right)\text{-d}_{\text{E2Fm}}\cdot\text{[E2Fm]} \end{matrix}$ |
| $\begin{matrix} \frac{\text{d[E2Fp]}}{\text{dt}}\text{=k}_{\text{E2Fp}}\cdot\text{[E2Fm]+}\frac{\text{k}_{\text{RBP1}}\cdot\text{[CD]}\cdot\text{[RE]}}{\text{K}_{\text{CD}}\text{+[RE]}}\text{ +}\frac{\text{k}_{\text{RBP2}}\cdot\text{[CE]}\cdot\text{[RE]}}{\text{K}_{\text{CE}}\text{+[RE]}} \\ \text{-k}_{\text{RE}}\cdot\text{[RB]}\cdot\text{[E2Fp]-}\left(\frac{\text{K}_{\text{RCA}}+\text{[CA]}}{\text{K}_{\text{RCA}}}\right)\cdot\text{d}_{\text{E2Fp}}\cdot\text{[E2Fp]} \end{matrix}$ |
| $\frac{\text{d[CA]}}{\text{dt}}\text{=k}_{\text{CA}}\cdot\frac{\text{[E2Fp]}}{\text{K}_{\text{CAEF}}\text{+[E2Fp]}}-\text{d}_{\text{CA}}\cdot\text{[CA]}$ |
| $\frac{\text{d[CD]}}{\text{dt}}\text{=k}_{\text{CD}}\cdot\frac{\text{[MYC]}}{\text{K}_{\text{MCCD}}\text{+[MYC]}}\text{+k}_{\text{CDS}}\cdot\frac{\text{[S]}}{\text{K}_{\text{S}}+\text{[S]}}\text{-d}_{\text{CD}}\cdot\text{[CD]}$ |
| $\frac{\text{d[CE]}}{\text{dt}}\text{=k}_{\text{CE}}\cdot\frac{\text{[E2Fp]}}{\text{K}_{\text{EF}}\text{+[E2Fp]}}-\text{d}_{\text{CE}}\cdot\text{[CE]}$ |
| $\begin{matrix} \frac{\text{d[RB]}}{\text{dt}}\text{=k}_{\text{RB}}\text{+k}_{\text{RBDP}}\cdot\frac{\text{[RP]}}{\text{K}_{\text{RP}}\text{+[RP]}}\text{-k}_{\text{RE}}\cdot\text{[RB]}\cdot\text{[E2Fp]-k}_{\text{RBP1}}\cdot\frac{\text{[CD]}\cdot\text{[RB]}}{\text{K}_{\text{CD}}\text{+[RB]}} \\ \text{-k}_{\text{RBP2}}\cdot\frac{\text{[CE]}\cdot\text{[RB]}}{\text{K}_{\text{CE}}\text{+[RB]}}\text{-d}_{\text{RB}}\cdot\text{[RB]} \end{matrix}$ |
| $\begin{matrix} \frac{\text{d[RP]}}{\text{dt}}\text{=k}_{\text{RBP1}}\cdot\frac{\text{[CD]}\cdot\text{[RB]}}{\text{K}_{\text{CD}}\text{+[RB]}}+\text{k}_{\text{RBP2}}\cdot\frac{\text{[CE]}\cdot\text{[RB]}}{\text{K}_{\text{CE}}\text{+[RB]}}-\text{k}_{\text{RBDP}}\cdot\frac{\text{[RP]}}{\text{K}_{\text{RP}}\text{+[RP]}}+\text{k}_{\text{RBP1}}\cdot\frac{\text{[CD]}\cdot\text{[RE]}}{\text{K}_{\text{CD}}\text{+[RE]}} \\ +\text{k}_{\text{RBP2}}\cdot\frac{\text{[CE]}\cdot\text{[RE]}}{\text{K}_{\text{CE}}\text{+[RE]}}\text{-d}_{\text{RP}}\cdot\text{[RP]} \end{matrix}$ |
| $\frac{\text{d[RE]}}{\text{dt}}\text{=k}_{\text{RE}}\cdot\text{[RB]}\cdot[\text{E2Fp}]-\text{k}_{\text{RBP1}}\cdot\frac{\text{[CD]}\cdot\text{[RE]}}{\text{K}_{\text{CD}}\text{+[RE]}}\text{-k}_{\text{RBP2}}\cdot\frac{\text{[CE]}\cdot\text{[RE]}}{\text{K}_{\text{CE}}\text{+[RE]}}\text{-d}_{\text{RE}}\cdot\text{[RE]}$ |
| $\frac{\text{d[R]}}{\text{dt}}\text{=k}_{\text{RMC}}\cdot\frac{\text{[MYC]}}{\text{K}_{\text{RMC}}\text{+[MYC]}}\text{-d}_{\text{R}}\cdot\text{[R]}$ |
| **Duplicated E2F model** |
| $\frac{\text{d[MYC]}}{\text{dt}}\text{=ke}_{\text{MC}}+\text{k}_{\text{MC}}\cdot\frac{\text{[S]}}{\text{K}_{\text{S}}\text{+[S]}}\text{-d}_{\text{MC}}\cdot\text{[MYC]}$ |
| $\begin{matrix} \frac{\text{d[E2Fm]}}{\text{dt}}\text{=}\left(\text{k}_{\text{S}}\cdot\frac{\text{[S]}}{\text{K}_{\text{S}}\text{+[S]}}\text{+k}_{\text{E2Fm}}\cdot\frac{\text{[MYC]}}{\text{K}_{\text{MC}}\text{+[MYC]}}\cdot\text{ }\frac{\text{[E2Fp]}+[\text{E2F'p]}}{\text{K}_{\text{EF}}\text{+[E2Fp]}+[\text{E2F'p]}}\text{+k}_{\text{b}}\cdot\frac{\text{[MYC]}}{\text{K}_{\text{MC1}}\text{+[MYC]}}\right) \\ \cdot\left(\frac{\text{K}_{\text{R}}}{\text{K}_{\text{R}}\text{+[R]}}\right)\text{-d}_{\text{E2Fm}}\cdot\text{[E2Fm]} \end{matrix}$ |
| $\begin{matrix} \frac{\text{d[E2Fp]}}{\text{dt}}\text{=k}_{\text{E2Fp}}\cdot\text{[E2Fm]+}\frac{\text{k}_{\text{RBP1}}\cdot\text{[CD]}\cdot\text{[RE]}}{\text{K}_{\text{CD}}\text{+[RE]}}\text{ +}\frac{\text{k}_{\text{RBP2}}\cdot\text{[CE]}\cdot\text{[RE]}}{\text{K}_{\text{CE}}\text{+[RE]}} \\ \text{-k}_{\text{RE}}\cdot\text{[RB]}\cdot\text{[E2Fp]-}\left(\frac{\text{K}_{\text{RCA}}+\text{[CA]}}{\text{K}_{\text{RCA}}}\right)\cdot\text{d}_{\text{E2Fp}}\cdot\text{[E2Fp]} \end{matrix}$ |
| $\frac{\text{d[CA]}}{\text{dt}}\text{=k}_{\text{CA}}\cdot\frac{\text{[E2Fp]}+[\text{E2F'p]}}{\text{K}_{\text{CAEF}}\text{+[E2Fp]}+[\text{E2F'p]}}-\text{d}_{\text{CA}}\cdot\text{[CA]}$ |
| $\frac{\text{d[CD]}}{\text{dt}}\text{=k}_{\text{CD}}\cdot\frac{\text{[MYC]}}{\text{K}_{\text{MCCD}}\text{+[MYC]}}\text{+k}_{\text{CDS}}\cdot\frac{\text{[S]}}{\text{K}_{\text{S}}+\text{[S]}}\text{-d}_{\text{CD}}\cdot\text{[CD]}$ |
| $\frac{\text{d[CE]}}{\text{dt}}\text{=k}_{\text{CE}}\cdot\frac{\text{[E2Fp]}+[\text{E2F'p]}}{\text{K}_{\text{EF}}\text{+[E2Fp]}+[\text{E2F'p]}}-\text{d}_{\text{CE}}\cdot\text{[CE]}$ |
| $\begin{matrix} \frac{\text{d[RB]}}{\text{dt}}\text{=k}_{\text{RB}}\text{+k}_{\text{RBDP}}\cdot\frac{\text{[RP]}}{\text{K}_{\text{RP}}\text{+[RP]}}\text{-k}_{\text{RE}}\cdot\text{[RB]}\cdot(\text{[E2Fp]}+[\text{E2F'p]})\text{-k}_{\text{RBP1}}\cdot\frac{\text{[CD]}\cdot\text{[RB]}}{\text{K}_{\text{CD}}\text{+[RB]}} \\ \text{-k}_{\text{RBP2}}\cdot\frac{\text{[CE]}\cdot\text{[RB]}}{\text{K}_{\text{CE}}\text{+[RB]}}\text{-d}_{\text{RB}}\cdot\text{[RB]} \end{matrix}$ |
| $\begin{matrix} \frac{\text{d[RP]}}{\text{dt}}\text{=k}_{\text{RBP1}}\cdot\frac{\text{[CD]}\cdot\text{[RB]}}{\text{K}_{\text{CD}}\text{+[RB]}}+\text{k}_{\text{RBP2}}\cdot\frac{\text{[CE]}\cdot\text{[RB]}}{\text{K}_{\text{CE}}\text{+[RB]}}-\text{k}_{\text{RBDP}}\cdot\frac{\text{[RP]}}{\text{K}_{\text{RP}}\text{+[RP]}}+\text{k}_{\text{RBP1}}\cdot\frac{\text{[CD]}\cdot\text{[RE]}}{\text{K}_{\text{CD}}\text{+[RE]}} \\ +\text{k}_{\text{RBP2}}\cdot\frac{\text{[CE]}\cdot\text{[RE]}}{\text{K}_{\text{CE}}\text{+[RE]}}+\text{k}_{\text{RBP1}}\cdot\frac{\text{[CD]}\cdot\text{[RE']}}{\text{K}_{\text{CD}}\text{+[RE']}}+\text{k}_{\text{RBP2}}\cdot\frac{\text{[CE]}\cdot\text{[RE']}}{\text{K}_{\text{CE}}\text{+[RE']}}\text{-d}_{\text{RP}}\cdot\text{[RP]} \end{matrix}$ |
| $\frac{\text{d[RE]}}{\text{dt}}\text{=k}_{\text{RE}}\cdot\text{[RB]}\cdot[\text{E2Fp}]-\text{k}_{\text{RBP1}}\cdot\frac{\text{[CD]}\cdot\text{[RE]}}{\text{K}_{\text{CD}}\text{+[RE]}}\text{-k}_{\text{RBP2}}\cdot\frac{\text{[CE]}\cdot\text{[RE]}}{\text{K}_{\text{CE}}\text{+[RE]}}\text{-d}_{\text{RE}}\cdot\text{[RE]}$ |
| $\frac{\text{d[R]}}{\text{dt}}\text{=k}_{\text{RMC}}\cdot\frac{\text{[MYC]}}{\text{K}_{\text{RMC}}\text{+[MYC]}}\text{-d}_{\text{R}}\cdot\text{[R]}$ |
| $\begin{matrix} \frac{\text{d[E2F'm]}}{\text{dt}}\text{=}\left(\text{k}_{\text{S'}}\cdot\frac{\text{[S]}}{\text{K}_{\text{S}}\text{+[S]}}\text{+k}_{\text{E2F'm}}\cdot\frac{\text{[MYC]}}{\text{K}_{\text{MC}}\text{+[MYC]}}\cdot\text{ }\frac{\text{[E2Fp]}+[\text{E2F'p]}}{\text{K}_{\text{EF}}\text{+[E2Fp]}+[\text{E2F'p]}}\text{+k}_{\text{b'}}\cdot\frac{\text{[MYC]}}{\text{K}_{\text{MC1}}\text{+[MYC]}}\right) \\ \cdot\left(\frac{\text{K}_{\text{R}}}{\text{K}_{\text{R}}\text{+[R]}}\right)\text{-d}_{\text{E2F'm}}\cdot\text{[E2F'm]} \end{matrix}$ |
| $\begin{matrix} \frac{\text{d[E2F'p]}}{\text{dt}}\text{=k}_{\text{E2F'p}}\cdot\text{[E2F'm]+}\frac{\text{k}_{\text{RBP1}}\cdot\text{[CD]}\cdot\text{[RE']}}{\text{K}_{\text{CD}}\text{+[RE']}}\text{ +}\frac{\text{k}_{\text{RBP2}}\cdot\text{[CE]}\cdot\text{[RE']}}{\text{K}_{\text{CE}}\text{+[RE']}} \\ \text{-k}_{\text{RE}}\cdot\text{[RB]}\cdot\text{[E2F'p]-}\left(\frac{\text{K}_{\text{RCA}}+\text{[CA]}}{\text{K}_{\text{RCA}}}\right)\cdot\text{d}_{\text{E2F'p}}\cdot\text{[E2F'p]} \end{matrix}$ |
| $\frac{\text{d[RE']}}{\text{dt}}\text{=k}_{\text{RE}}\cdot\text{[RB]}\cdot[\text{E2F'p}]-\text{k}_{\text{RBP1}}\cdot\frac{\text{[CD]}\cdot\text{[RE']}}{\text{K}_{\text{CD}}\text{+[RE']}}\text{-k}_{\text{RBP2}}\cdot\frac{\text{[CE]}\cdot\text{[RE']}}{\text{K}_{\text{CE}}\text{+[RE']}}\text{-d}_{\text{RE}}\cdot\text{[RE']}$ |

## Table S3. Model parameters

| **Parameter** | **Base Value** | **Range** | **Description** |
| --- | --- | --- | --- |
| $\text{ke}_{\text{MC}}$ | 0.01 μM/h | [10^-2^ 10^1^] | MYC synthesis rate (external input/mutation) |
| $\text{k}_{\text{MC}}$ | 0.01 μM/h | [10^-2^ 10^1^] | MYC synthesis rate (serum) |
| $\text{d}_{\text{MC}}$ | 0.7 /h | [10^-2^ 10^1^] | MYC decay constant |
| $\text{k}_{\text{b(')}}$ | 0.15 μM/h | [10^-2^ 10^1^] | E2F(’)m synthesis rate (MYC) |
| $\text{k}_{\text{S(')}}$ | 0.05 μM/h | [10^-2^ 10^1^] | E2F(’)m synthesis rate (serum) |
| $\text{k}_{\text{CD}}$ | 0.03 μM/h | [10^-2^ 10^1^] | CYC_D_ synthesis rate (MYC) |
| $\text{k}_{\text{CDS}}$ | 0.45 μM/h | [10^-2^ 10^1^] | CYC_D_ synthesis rate (serum) |
| $\text{d}_{\text{CD}}$ | 1.5 /h | [10^-2^ 10^1^] | CYC_D_ decay constant |
| $\text{k}_{\text{RBP1}}$ | 18 /h | [10^-1^ 10^2^] | RB phosphorylation rate (CYC_D_/CDK_4,6_) |
| $\text{k}_{\text{E2F(')m}}$ | 0.40 μM/h | [10^-2^ 10^1^] | E2F(‘)m synthesis rate (MYC/E2F feedback) |
| $\text{d}_{\text{E2F(')m}}$ | 0.25 /h | [10^-2^ 10^1^] | E2F(‘)m decay constant |
| $\text{k}_{\text{E2F(')p}}$ | 0.40 /h | [10^-2^ 10^1^] | E2F(‘) translation rate |
| $\text{d}_{\text{E2F(')p}}$ | 0.35 /h | [10^-2^ 10^1^] | E2F(‘)p decay constant |
| $\text{k}_{\text{RBP2}}$ | 18 /h | [10^-1^ 10^2^] | RB phosphorylation rate (CYC_E_/CDK_2_) |
| $\text{k}_{\text{CE}}$ | 0.35 μM/h | [10^-2^ 10^1^] | CE synthesis rate (E2Fp) |
| $\text{d}_{\text{CE}}$ | 1.5 /h | [10^-2^ 10^1^] | CYC_E_ decay constant |
| $\text{k}_{\text{CA}}$ | 0.10 μM/h | [10^-2^ 10^1^] | CYC_A_ synthesis rate (E2Fp) |
| $\text{d}_{\text{CA}}$ | 0.10 /h | [10^-2^ 10^1^] | CYC_A_ decay constant |
| $\text{k}_{\text{R}}$ | 0.10 μM/h | [10^-2^ 10^1^] | Repressor synthesis rate |
| $\text{d}_{\text{R}}$ | 0.10/h | [10^-2^ 10^1^] | Repressor decay constant |
| $\text{k}_{\text{RB}}$ | 0.18 μM/h | *Fixed* | RB synthesis rate |
| $\text{d}_{\text{RB}}$ | 0.06 /h | *Fixed* | RB decay constant |
| $\text{k}_{\text{RE}}$ | 180 /(μM*h) | *Fixed* | RB-E2F formation rate |
| $\text{d}_{\text{RE}}$ | 0.03 /h | *Fixed* | RB-E2F decay constant |
| $\text{k}_{\text{RBDP}}$ | 3.6 μM/h | *Fixed* | RB dephosphorylation rate |
| $\text{d}_{\text{RP}}$ | 0.70 /h | *Fixed* | RP decay constant for 60 minute half-life of p130 |
| $\text{K}_{\text{S}}$ | 0.5 % | *Fixed* | Half-maximal serum concentration |
| $\text{K}_{\text{MC}}$ | 0.15 μM | *Fixed* | Half-maximal MYC (E2Fm autoregulation) |
| $\text{K}_{\text{MC1}}$ | 2.5 μM | *Fixed* | Half-maximal MYC (E2Fp-independent E2Fm regulation) |
| $\text{K}_{\text{MCCD}}$ | 0.15 μM | *Fixed* | Half-maximal MYC (CYC_D_ synthesis) |
| $\text{K}_{\text{EF}}$ | 0.15 μM | *Fixed* | Half-maximal E2Fp (E2F autoregulation) |
| $\text{K}_{\text{CD}}$ | 0.92 μM | *Fixed* | Half-maximal CYC_D_ (RB phosphorylation) |
| $\text{K}_{\text{CE}}$ | 0.92 μM | *Fixed* | Half-maximal CYC_E_ (RB phosphorylation) |
| $\text{K}_{\text{RP}}$ | 0.01 μM | *Fixed* | Michaelis-Menten constant (RB dephosphorylation) |
| $\text{K}_{\text{CAEF}}$ | 0.15 μM | *Fixed* | Half-maximal E2Fp (CYC_A_ synthesis) |
| $\text{K}_{\text{RCA}}$ | 0.50 μM | *Fixed* | Binding constant E2Fp:CYC_A_ complex |
| $\text{K}_{\text{R}}$ | 1.0 μM | *Fixed* | Half-maximal R for E2Fm repression |
| $\text{K}_{\text{RMC}}$ | 50 μM | *Fixed* | Half-maximal MYC (R synthesis) |

# MATERIALS AND METHODS

# Parameter search algorithm

To examine how these different dynamics can be reconciled in a common network, we developed a search algorithm to identify parameters that support each. To simplify the analysis, we allowed synthesis and degradation rates to vary (Table S3) while fixing a subset of parameters unlikely to be subject to external control in the different biological contexts examined here. For one iteration of the algorithm, values of free parameters are initialized randomly from a log-uniform distribution spanning at least three orders of magnitude. Each parameter range was determined by restricting them to a physiologically relevant range (e.g. mRNA and protein half-lives) and/or were adjusted so that they included a previously established base value for each parameter. We simulated time courses of several network components in response to serum (to examine bistability or adaptation) or MYC (to examine biphasic dose response). Each response is scored by an objective function that measures the corresponding temporal (adaptive) or dose (hysteretic and biphasic) response (Figure S1*A*).

For each round of simulation, the score was compared against a pre-defined minimum threshold. If the score exceeded the threshold, the corresponding parameter set was defined as a solution for the particular function. Otherwise, the parameter set was permutated (“mutated”) in a probabilistic fashion before another round of simulation and evaluation (see pseudo-code below). The probability (prob) of mutating any given parameter within a set is 0.40; Mutations were created by drawing from the normal distribution ~N(0,1) and adding this to the logged value of the current parameter. If the score improved within the last consecutive 100 mutations, the parameter set was permitted to engage subsequent rounds of mutation and scoring. This procedure was repeated until the score exceeded the threshold. If the score did not improve after 100 mutations, however, the iteration was terminated without a solution. Typically, 10,000 iterations of the algorithm were performed for each dynamic task (single and dual).

This approach to parameter searching possesses two desirable characteristics. By defining solutions as parameter sets which exceed an objective function score, the algorithm reports sub-optimal (but nevertheless valid) solutions that are often discarded in place of higher scoring ones in more traditional evolutionary approaches (i.e. genetic algorithm). At the same time, the algorithm can permute parameters in a probabilistic fashion and retain parameter sets with the potential to climb a fitness landscape towards a solution. This is distinct from ‘brute-force’ random scanning methods, which are impractical for probing high-dimensional spaces. In particular, in order to survey P points of each parameter dimension D, one would be required to test exactly P^D^ parameter sets. In the context of our model, such a search would easily become infeasible: If P=3 (i.e., search three points in the 3-log range for each parameter) then this requires 3^19^ = 10^9^ or one billion distinct parameter sets.

- - - **Pseudo-code for a parameter search iteration**

threshold <- minimum objective score

isFinished <- false

iteration <- 0

topScore <- 0

M <- randomly initialize parameter set

S <- Score M

while isFinished is false and iteration < 100

Mmutated <- Mutate M

Smutated <- Score Mmutated

if S < Smutated

M <- Mmutated

topScore <- Smutated

iteration <- 0

else

iteration <- iteration + 1

endif

if S > threshold

isFinished <- true

endif

endwhile

return M

- - - **Pseudo-code for Mutate**

bounds <- bounding values for parameters

prob <- mutation probability

sigma <- normal distribution standard deviation

for each parameter p in vector

valid <- false

r <- random ~Uniform(0 1)

if r < prob

while valid is false

ptemp <- p + ~Normal(0,sigma)

if ptemp is within bounds

p <- ptemp

valid <- true

endif

endwhile

end

endfor

# Definitions

## Tension

We define tension between two dynamic tasks. Consider a network with n parameters ($\overrightarrow{x}=[x_{1},x_{2},x_{3},...,x_{n}]$). For each single task (*i*), formulate a criterion in the form of an objective function:

$$O_{i}:R^{n}\to R$$

The solution space for each single task ($S_{i}$) is the subset of parameter space where the value objective $O_{i}$ exceeds a minimum value ($\varepsilon_{i}$):

$S_{i}$ will be a function of the particular network topology and the range of values of each parameter will be subject to boundary conditions representing physiologically plausible range.

Implementations of multi-objective, stochastic optimization have often used an objective defined by the weighted-sum of individual objectives which we have also employed here according to:

$$O_{ij}=\omega_{i}\cdot O_{i}+\omega_{j}\cdot O_{j}$$

where the weights (ω) in this study are equal to unity. The results of this approach to identifying dual solutions are denoted “Dual^Additive^” in Figure S2 and Figure S3. We also defined a objective function assuming independent contributions from each single task objective denoted “Dual^Product^ ”:

$$O_{ij}=O_{i}\cdot O_{j}$$

As an independent validation of this approach, we performed a search for dual solutions using the set of single solutions for either task as a starting point in place of randomly initialized parameters. In this way, single task objectives are considered sequentially (denoted “Dual^single task IC^”, Figure S2 and Figure S3), mimicking the sequential addition of tasks onto a network. In all cases where a composite objective is employed, each individual objective must exceed the required threshold score in order to be deemed a “dual”. Each implementation of the composite objective gave similar distributions of solution parameters.

Several challenges are faced when trying to compare solution spaces including the high-dimensionality of the system, the interdependent nature of parameters, and the possibility of solution space that is disjoint rather than simply connected. Thus, as a first approximation, we assume that each parameter contributes independently and make no assumptions regarding its distribution in parameter space. Thus, we calculate the median of each parameter value for the solution set and define the tension (T) between two solutions spaces associated with two tasks (i and j) as:

$$\begin{matrix} T_{ij}=\sum_{p=1}^{n} \omega_{p}\cdot\left|\log\left(\frac{median(x_{p}^{i})}{median(x_{p}^{j})}\right)\right| \\ \end{matrix}$$

where *n* is the number of parameters , and ω*_p_* is a weighting factor that can be included if there is *a priori* knowledge regarding the relative importance of the parameter on system dynamics. In this report, we assign equal weights to all parameters such that ω*_p_=1/n*.

## Kullback-Leibler divergence

For each parameter dimension, the distribution of the solutions selected for a single dynamics (hysteretic, biphasic, or adaptive) is fitted with a nonparametric kernel-smoothing distribution. The Kullback-Leibler (KL) divergence between the solution distributions generating two distinct network dynamics is calculated using the fitted probability density functions.

The KL divergence between p with respect to q is defined as:

$$D(p||q)=\int p(x)\log\frac{p(x)}{q(x)}dx$$

## Accessibility

One measure of robustness is the fraction of parameters surveyed that generate a dynamic, providing a measure of the effective “size” of the solution space. Our parameter search algorithm is able to perform a local, random search in parameter space and is biased towards those that increase the objective score. Intuitively, a “smooth” objective landscape will allow the algorithm to converge to a maximum more rapidly and easily than a “rugged” landscape with many local maxima/minima.

The results of our search algorithm can be used to infer the size and nature of the landscape surrounding the solution space for a particular task: Under equivalent conditions (i.e. network topology, algorithm parameters, total iterations, and model parameters), the number of solutions can be used to calculate the “accessibility” of a solution space, which in the case of dual solutions is:

$A_{ij}=\frac{N^{ij}}{N^{i}+N^{j}}$,

where *N^ij^* is the number of dual solutions that occur within the solutions for each single task (i and j) and *N^i^* and *N^j^* are the total number of single task solutions.

## Resiliency

Another measure of robustness concerns the ability of an individual solution to maintain its performance in the face of parameter perturbation. In this report we tested solutions from each task (single and dual) that were in close proximity to the median value for each respective solution space. Selecting solutions based upon different criteria (random, highest scoring, lowest scoring) had no significant impact on the results. For each solution, each of its n parameters was perturbed by selecting a new value drawn from a normal distribution centered on *x_p_^original^* to get *x_p_^perturbed^* by using the “Mutate” algorithm defined above. Total parameter variation for a perturbed parameter set is defined as. A perturbed parameter set for a particular task (*i*) is resilient if it retains at least 10% of the original parameter objective function score. That is,

.

Varying the threshold between 0.5 and 0.01 did not significantly impact the change in resiliency between single and dual solutions. The resilient fraction reported in Figures 2*E*, 3*E*, and 4*E* of the main text represent the results of 10,000 perturbations to each individual solution.

# REFERENCES

1. Schulze A, Zerfass K, Spitkovsky D, Middendorp S, Berges J, et al. (1995) Cell cycle regulation of the cyclin A gene promoter is mediated by a variant E2F site. Proc Natl Acad Sci U S A 92: 11264-11268.

2. Xu M, Sheppard KA, Peng CY, Yee AS, Piwnica-Worms H (1994) Cyclin A/CDK2 binds directly to E2F-1 and inhibits the DNA-binding activity of E2F-1/DP-1 by phosphorylation. Mol Cell Biol 14: 8420-8431.

3. Mudryj M, Devoto SH, Hiebert SW, Hunter T, Pines J, et al. (1991) Cell cycle regulation of the E2F transcription factor involves an interaction with cyclin A. Cell 65: 1243-1253.

4. Krek W, Xu G, Livingston DM (1995) Cyclin A-kinase regulation of E2F-1 DNA binding function underlies suppression of an S phase checkpoint. Cell 83: 1149-1158.

5. Krek W, Ewen ME, Shirodkar S, Arany Z, Kaelin WG, Jr., et al. (1994) Negative regulation of the growth-promoting transcription factor E2F-1 by a stably bound cyclin A-dependent protein kinase. Cell 78: 161-172.

6. Zhang L, Wang C (2006) F-box protein Skp2: a novel transcriptional target of E2F. Oncogene 25: 2615-2627.

7. Marti A, Wirbelauer C, Scheffner M, Krek W (1999) Interaction between ubiquitin-protein ligase SCFSKP2 and E2F-1 underlies the regulation of E2F-1 degradation. Nat Cell Biol 1: 14-19.

8. Mason SL, Loughran O, La Thangue NB (2002) p14(ARF) regulates E2F activity. Oncogene 21: 4220-4230.

9. Martelli F, Hamilton T, Silver DP, Sharpless NE, Bardeesy N, et al. (2001) p19ARF targets certain E2F species for degradation. Proc Natl Acad Sci U S A 98: 4455-4460.

10. Eymin B, Karayan L, Séité P, Brambilla C, Brambilla E, et al. (2001) Human ARF binds E2F1 and inhibits its transcriptional activity. Oncogene 20: 1033--1041.

11. Datta A, Sen J, Hagen J, Korgaonkar CK, Caffrey M, et al. (2005) ARF directly binds DP1: interaction with DP1 coincides with the G1 arrest function of ARF. Mol Cell Biol 25: 8024--8036.

12. Datta A, Nag A, Raychaudhuri P (2002) Differential regulation of E2F1, DP1, and the E2F1/DP1 complex by ARF. Mol Cell Biol 22: 8398--8408.

13. Komori H, Enomoto M, Nakamura M, Iwanaga R, Ohtani K (2005) Distinct E2F-mediated transcriptional program regulates p14ARF gene expression. EMBO J 24: 3724-3736.

14. Bates S, Phillips AC, Clark PA, Stott F, Peters G, et al. (1998) p14ARF links the tumour suppressors RB and p53. Nature 395: 124--125.

15. Zindy F, Eischen CM, Randle DH, Kamijo T, Cleveland JL, et al. (1998) Myc signaling via the ARF tumor suppressor regulates p53-dependent apoptosis and immortalization. Genes Dev 12: 2424--2433.

16. Woods K, Thomson JM, Hammond SM (2007) Direct regulation of an oncogenic micro-RNA cluster by E2F transcription factors. J Biol Chem 282: 2130--2134.

17. Sylvestre Y, Guire VD, Querido E, Mukhopadhyay UK, Bourdeau V, et al. (2007) An E2F/miR-20a autoregulatory feedback loop. J Biol Chem 282: 2135--2143.

18. Pickering MT, Stadler BM, Kowalik TF (2008) miR-17 and miR-20a temper an E2F1-induced G1 checkpoint to regulate cell cycle progression. Oncogene.

19. O'Donnell KA, Wentzel EA, Zeller KI, Dang CV, Mendell JT (2005) c-Myc-regulated microRNAs modulate E2F1 expression. Nature 435: 839--843.

20. Wong JV, Yao G, Nevins JR, You L (2011) Viral-Mediated Noisy Gene Expression Reveals Biphasic E2f1 Response to MYC. Mol Cell 41: 275-285.

21. Tedesco D, Lukas J, Reed SI (2002) The pRb-related protein p130 is regulated by phosphorylation-dependent proteolysis via the protein-ubiquitin ligase SCF(Skp2). Genes Dev 16: 2946-2957.

22. Yao G, Lee TJ, Mori S, Nevins JR, You L (2008) A bistable Rb-E2F switch underlies the restriction point. Nat Cell Biol 10: 476--482.

23. Arora JS, Marler RT (2004) Survey of multi-objective optimization methods for engineering. Structural and Multidisciplinary Optimization 26: 369-395.

24. Cover TM, Thomas JA (2006) Elements of information theory. 2nd ed. Hoboken, N.J.: Wiley-Interscience. pp. xxiii, 748 p.

25. Ashall L, Horton CA, Nelson DE, Paszek P, Harper CV, et al. (2009) Pulsatile stimulation determines timing and specificity of NF-kappaB-dependent transcription. Science 324: 242-246.

26. Meir E, von Dassow G, Munro E, Odell GM (2002) Robustness, flexibility, and the role of lateral inhibition in the neurogenic network. Curr Biol 12: 778-786.

27. Santos SD, Verveer PJ, Bastiaens PI (2007) Growth factor-induced MAPK network topology shapes Erk response determining PC-12 cell fate. Nat Cell Biol 9: 324-330.

28. Lahav G, Rosenfeld N, Sigal A, Geva-Zatorsky N, Levine AJ, et al. (2004) Dynamics of the p53-Mdm2 feedback loop in individual cells. Nat Genet 36: 147-150.

29. Batchelor E, Loewer A, Mock C, Lahav G (2011) Stimulus-dependent dynamics of p53 in single cells. Mol Syst Biol 7: 488.
